# Osteoarthritis, coronary artery disease, and myocardial infarction: A mendelian randomization study

**DOI:** 10.3389/fcvm.2022.892742

**Published:** 2022-08-25

**Authors:** Huiqing Xu, Yuxiao Ling, Han Jiang, Yingjun Li, Minmin Jiang

**Affiliations:** ^1^School of Public Health, Hangzhou Medical College, Hangzhou, China; ^2^Key Laboratory of Pollution Exposure and Health Intervention of Zhejiang Province, Shulan International Medical College, Zhejiang Shuren University, Hangzhou, China

**Keywords:** coronary artery disease, myocardial infarction, osteoarthritis, Mendelian randomization, protective effect

## Abstract

**Background:**

Observational studies indicate that osteoarthritis (OA) and coronary artery disease (CAD), as well as myocardial infarction (MI), are often diagnosed as comorbid diseases. We performed a bidirectional Mendelian randomization (MR) study to demonstrate whether there is a causal relationship between OA, CAD, and MI.

**Methods:**

We extracted single nucleotide polymorphisms (SNPs) related to OA in the Genetics of Osteoarthritis (GO) Consortium as instrumental variables to assess whether OA is associated with CAD and MI in the CARDIoGRAMplusC4D 1,000 Genomes genome-wide association study (GWAS). In the reverse MR, we used CAD-associated and MI-associated SNPs to the GWAS of OA to analyze their causality. These GWASs included 766,690 individuals of OA, 184,305 individuals of CAD, and 166,065 individuals of MI. MR was conducted using several methods, including the inverse variance weighted (IVW) method, the weighted median method, the MR-Egger method, and the MR-Pleiotropy RESidual Sum and Outlier (MR-PRESSO) method.

**Results:**

The forward causal effect of OA on CAD and MI was not observed. In reverse analysis, no causal effect was discovered for CAD on the risk of OA. Notably, we observed a causal association between MI and total OA [IVW odds ratio (OR) = 0.95, 95% CI = 0.93, 0.98, *P* = 4E−04] and spine OA (IVW OR = 0.92, 95% CI = 0.88, 0.97, *P* = 0.001) but a null association between MI and knee OA, hip OA, hand OA, and thumb OA.

**Conclusion:**

This MR study identifies a potentially protective effect of genetically predicted MI on total and spine OA risks.

## Introduction

Cardiovascular diseases (CVDs), including coronary artery disease (CAD) and myocardial infarction (MI), and musculoskeletal disorders, including osteoarthritis (OA), are the main leading causes of mortality and disability worldwide (1, 2). They are often diagnosed as comorbid diseases and impair the quality of life significantly, especially among older adults (3). The possible association between CVDs and OA may be due to systemic inflammation. Since CAD, MI, and OA share common risk factors, such as aging and obesity (4–6), the association between the two prevailing conditions was explored increasingly in epidemiological studies. Jonsson et al. reported that hand OA in older women was associated with coronary atherosclerosis in the population-based multidisciplinary study of aging in the older adult population of Reykjavik (7). The presence of OA significantly increased the risk of new CAD in older adults according to the findings from the Progetto Veneto Anziano Study Cohort after 4.4 years of follow-up (8). The results from a systematic review and meta-analysis of population-based studies demonstrated that OA was related to a 31% increased risk of MI in the general population (9). However, no significantly higher risk of MI was detected in people with OA compared with the non-OA group from another systematic review and meta-analysis (10). The exact relationships between OA with CAD and MI remain unclear due to the contradictory evidence.

On the contrary, conclusions derived from observational studies were potentially biased due to residual confounding and reverse causality (11). Moreover, the cause-effect relationship between two diseases cannot be demonstrated by randomized controlled trials (RCTs) because of ethical and practical reasons (12). Mendelian randomization (MR) is a novel method that follows the law of independent assortment and uses genetic variants as instrumental variables (IVs) to assess the causal effects of exposure on the outcome (13, 14). Since the genotype of an individual is determined at conception and cannot be changed, this method largely avoids the reverse causality between the genetic phenotype and the associated outcome.

In the present study, we attempted to verify whether OA was causally associated with CAD and MI and to investigate whether CAD and/or MI were causally correlated with OA by leveraging summary genome-wide association studies (GWASs) data. A two-sample bidirectional MR framework was used to disentangle the causality and the direction of this association.

## Methods

### Study population

We chose genetic variants and extracted summary statistics of OA and its phenotypes from the largest genome-wide association study (GWAS) on the Genetics of Osteoarthritis (GO) Consortium participants of mainly European ancestry (177,517 cases and 649,173 controls) (15). OA was defined by the GO based on either self-reported status, the hospital diagnosed, the International Classification of Diseases-10 (ICD-10) codes, or radiography defined by the TREAT-OA Consortium (16). The study population is given in Table 1.

**Table 1:** Baseline characteristics of the study population.

**Traits**	**Consortium**	**Sample size**	***N* cases**	***N* controls**	**Age, years**	**Women, % (*N*)**	**Number of SNP**	**Population studied**
CAD	CARDIoGRAMplusC4D	184,305	60,801	123,504	NA	NA	8,597,751	European (77%)
MI	CARDIoGRAMplusC4D	166,065	42,561	123,504	NA	NA	9,289,492	European (77%)
Total OA	Genetics of osteoarthritis consortium	826,690	177,517	649,173	58.4	54.0 (446,499)	25,924,626	European (99%)
Knee OA	Genetics of osteoarthritis consortium	396,054	62,497	333,557	59.6	52.0 (205,770)	22,173,239	European (99%)
Hip OA	Genetics of osteoarthritis consortium	353,388	36,445	316,943	59.8	50.6 (178,861)	18,871,781	European
Spine OA	Genetics of osteoarthritis consortium	333,950	28,372	305,578	58.3	50.3 (167,959)	19,360,900	European (98%)
Hand OA	Genetics of osteoarthritis consortium	303,782	20,901	282,881	59.4	49.6 (150,789)	15,712,743	European
Thumb OA	Genetics of osteoarthritis consortium	247,455	10,536	236,919	60.3	48.6 (120,309)	14,232,525	European

CAD, coronary artery disease; MI, myocardial infarction; OA, osteoarthritis; CARDIoGRAMplusC4D, Coronary ARtery DIsease Genome wide Replication and Meta-analysis plus The Coronary Artery Disease Genetics; NA, not available.

Genetic architectures of CAD were publicly available from a GWAS meta-analysis contributed to by the CARDIoGRAMplusC4D 1,000 Genomes, involving ~184,305 participants of mainly European ancestry (77%) (17). Summary statistics data of MI were obtained from the same GWAS, including 166,065 individuals, and used as a subgroup analysis. In the GWAS, CAD diagnosis included MI, acute coronary syndrome, chronic stable angina, or coronary stenosis >50%.

Ethical approval was not required in the present study as this was a secondary analysis of existing published data.

### Study design

**Figure 1:**
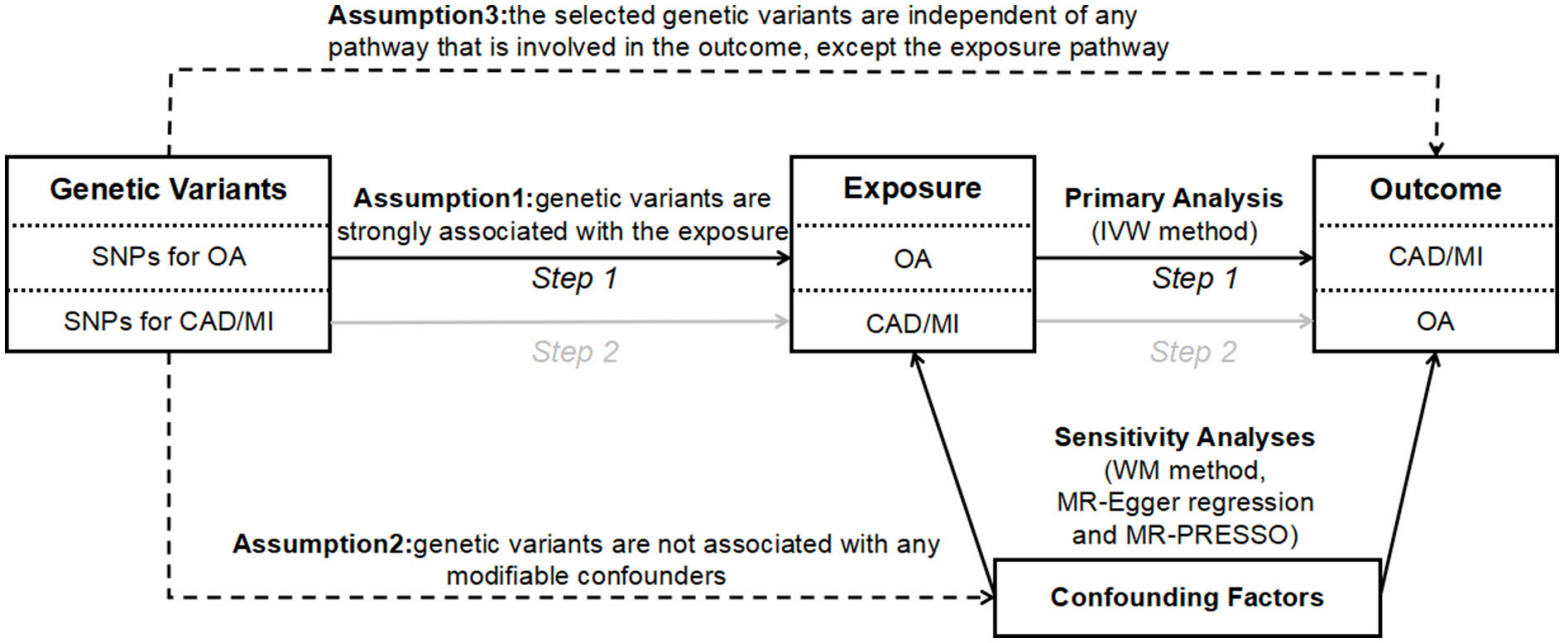
The design of bidirectional MR study. The solid paths are significant; dashed paths should not exist in the MR study. SNP, single nucleotide polymorphism; CAD, coronary artery disease; MI, myocardial infarction; OA, osteoarthritis; IVW, inverse variance weighted; WM, weighted median; MR-PRESSO, Mendelian randomization pleiotropy residual sum and outlier.

The design of the bidirectional MR study is shown in Figure 1. Briefly, the causal effects of OA on CAD and MI were first estimated. Then, the causal effects of CAD and MI on OA were analyzed. The genetic variants used as IVs must meet the following three stringent assumptions: (1) genetic variants are strongly associated with the exposures; (2) genetic variants are not associated with any modifiable confounders; and (3) the selected genetic variants are independent of any pathway that is involved in the outcome, except the exposure pathway (18). The genetic variants satisfying the second and third assumptions' mean independence from pleiotropy.

### Single nucleotide polymorphism selection

Single nucleotide polymorphisms (SNPs) identified as IVs must be strongly associated with the exposure. Thus, the selected *p-value* was at a genome-wide significant level (*p* < 5 × 10^−8^). Besides, we performed a linkage disequilibrium (LD) test on each SNP identified as IVs to prune the SNPs for pairs with *r*^2^ > 0.01 so as to ensure independence among the SNPs.

We searched all of the SNPs associated with exposure in the PhenoScanner database to identify whether they were associated with any modifiable confounders and outcomes (19). These satisfied the last two assumptions of IVs, that is, the genetic variants involved in the outcome only through the exposure pathway. We estimated the causal effects of the SNPs on outcomes. Proxy SNPs with strong linkage disequilibrium (*r*^2^ > 0.8) were used to replace SNPs not available in the outcome GWAS. If no proxy could be obtained, the SNP was excluded from our MR study. Finally, *R*^2^ and *F*-statistics were calculated to assess the strength of IVs (20). The details of instrument SNPs are given in Supplementary Tables 1–4.

### Mendelian randomization analysis

We conducted the MR analysis using several methods, including the inverse variance weighted (IVW) method, the weighted median (WM) method, the MR-Egger method, and the MR-Pleiotropy RESidual Sum and Outlier (MR-PRESSO) method. The IVW was performed as our primary MR analysis methodology to estimate the overall effect of genetically predicted exposure on outcome. The WM, MR-Egger, and MR-PRESSO methods were employed as additional sensitivity analyses to evaluate the two-sample MR assumptions (Figure 1). Specifically, the WM method provides an accurate estimate even when 50% of the genetic variants violate the core MR assumptions (21). The MR-Egger method may provide a correct estimate of causality, though no genetic variants satisfy the core MR assumptions (22). Besides, we introduced the MR-PRESSO method, which is a method for the detection and correction of outliers in IVW linear regression (23). Moreover, we used Cochran's Q statistics as a measure of heterogeneity (*P*-values for Cochran's *Q* < 0.05 suggest the existence of heterogeneity). We adopted the MR-Egger method to analyze the existence of directional pleiotropy, expressed by the size of the intercept (*P*-values for the MR-Egger method intercept < 0.05 suggest the existence of directional pleiotropy) (24). The “leave-one-out analysis” by removal of every single SNP at turn could assess the reliability of the results.

All of the analyses above were performed with the “TwoSampleMR” and “MR-PRESSO” packages in R software. The effect estimates of exposure on outcome were presented as odds ratios (ORs) and 95% CIs. Given the issue of multiple testing, the main results possessed statistical significance at *P*-values < 0.002 (0.05/24) after a Bonferroni correction.

## Results

### Causal effect of osteoarthritis and its phenotypes on coronary artery disease and myocardial infarction

After evaluating all the OA-associated SNPs through the PhenoScanner database and matching them with the summary data of SNP outcome (CAD or MI), we discovered that eight of them exhibited association with confounding factors (such as body mass index and/or waist-to-hip ratio), and one SNP associated with knee OA and one SNP associated with spine OA were not found in the outcome GWAS. Lastly, 16, 18, 36, 7, and 4 genetic variants with LD independence were taken as IVs for the total, knee, hip, hand, and thumb OA, respectively (Supplementary Table 1). F-statistics for IVs of OA were above the threshold of 10, suggesting that the IVs were strong instruments and reduced the bias of IV estimates.

As seen in Table 2, using the IVW method, total OA (OR = 0.93, 95% CI = 0.65, 1.32, *P* = 0.675) and each of its subtypes were not related to the risk of CAD. These findings were similar to the other MR estimates. Significant heterogeneity between the selected IVs of the total OA and the knee OA and CAD was observed (*P* < 0.05), but no heterogeneity was detected for the hip, hand, and thumb OA. The MR-Egger analysis did not suggest any directional pleiotropy for the IVs.

**Table 2:** MR results of the association between OA and CAD.

**Traits**	**No. of SNPs**	**Method**	**OR (95% CI)**	***P***	**Heterogeneity test**	**Pleiotropy test**
					**Cochran's Q (*P*^a^)**	***P* Intercept**
Total OA and CAD	16	IVW	0.93 (0.65, 1.32)	0.675	68.94 (** <0.001**)	
		WM	1.15 (0.89, 1.48)	0.289		
		MR Egger	1.01 (0.14, 7.23)	0.993		0.933
		MR-PRESSO (Outlier-corrected)	1.07 (0.83, 1.31)	0.585		
Knee OA and CAD	18	IVW	1.01 (0.88, 1.15)	0.903	31.78 (**0.016**)	
		WM	1.01 (0.88, 1.15)	0.937		
		MR Egger	1.49 (0.41, 5.40)	0.549		0.555
		MR-PRESSO (Outlier-corrected)	0.96 (0.86, 1.06)	0.434		
Hip OA and CAD	36	IVW	1.01 (0.95, 1.07)	0.860	45.90 (0.103)	
		WM	1.02 (0.95, 1.10)	0.535		
		MR Egger	0.99 (0.79, 1.24)	0.903		0.863
		MR-PRESSO (Outlier-corrected)	–	–		
Hand OA and CAD	7	IVW	1.06 (0.94, 1.20)	0.334	11.63 (0.071)	
		WM	1.05 (0.92, 1.19)	0.460		
		MR Egger	1.30 (0.53, 3.17)	0.589		0.672
		MR-PRESSO (Outlier-corrected)	–	–		
Thumb OA and CAD	4	IVW	1.08 (0.97, 1.19)	0.153	3.67 (0.300)	
		WM	1.06 (0.95, 1.19)	0.303		
		MR Egger	0.79 (0.22, 2.81)	0.746		0.675
		MR-PRESSO (Outlier-corrected)	–	–		

MR, Mendelian randomization; OA, osteoarthritis; CAD, coronary artery disease; SNPs, single nucleotide polymorphisms; OR, odds ratio; CI, confidence interval; IVW, inverse variance weighted; WM, weighted median; MR-PRESSO, MR pleiotropy residual sum and outlier.

a Bolded P represents heterogeneity.

Additionally, total OA (IVW OR = 0.95, 95% CI = 0.70, 1.29, *P* = 0.739) and its subtypes had no causal effect on the risk of MI, as seen in Table 3. Similar findings were obtained using the other MR estimate methods. No heterogeneity was detected by Cochran's *Q*-test for OA and its phenotypes, except for the total and knee OA. The MR-Egger analysis did not demonstrate any directional pleiotropy for the IVs.

**Table 3:** MR results of the association between OA and MI.

**Traits**	**No. of SNPs**	**Method**	**OR (95%CI)**	***P***	**Heterogeneity test**	**Pleiotropy test**
					**Cochran's Q (*P*^a^)**	***P* Intercept**
Total OA and MI	16	IVW	0.95 (0.70, 1.29)	0.739	43.74 (** <0.001**)	
		WM	1.00 (0.75, 1.32)	0.988		
		MR Egger	1.65 (0.30, 8.98)	0.572		0.526
		MR-PRESSO (Outlier-corrected)	1.13 (0.90, 1.36)	0.315		
Knee OA and MI	18	IVW	1.00 (0.87, 1.16)	0.999	31.10 (**0.019**)	
		WM	0.97 (0.83, 1.14)	0.737		
		MR Egger	1.02 (0.24, 4.26)	0.978		0.978
		MR-PRESSO (Outlier-corrected)	0.95 (0.83, 1.06)	0.361		
Hip OA and MI	35^b^	IVW	0.98 (0.93, 1.04)	0.538	35.04 (0.419)	
		WM	0.99 (0.91, 1.07)	0.756		
		MR Egger	0.97 (0.76, 1.22)	0.775		0.888
		MR-PRESSO (Outlier-corrected)	–	–		
Hand OA and MI	7	IVW	1.03 (0.93, 1.13)	0.593	6.30 (0.391)	
		WM	1.06 (0.93, 1.22)	0.357		
		MR Egger	1.41 (0.71, 2.80)	0.370		0.401
		MR-PRESSO (Outlier-corrected)	–	–		
Thumb OA and MI	4	IVW	1.00 (0.91, 1.11)	0.981	2.14 (0.544)	
		WM	1.02 (0.90, 1.14)	0.802		
		MR Egger	0.61 (0.21, 1.83)	0.474		0.471
		MR-PRESSO (Outlier-corrected)	–	–		

MR, Mendelian randomization; OA, osteoarthritis; MI, myocardial infarction; SNPs, single nucleotide polymorphisms; OR, odds ratio; CI, confidence interval; IVW, inverse variance weighted; WM, weighted median; MR-PRESSO, MR pleiotropy residual sum and outlier.

a Bolded P-value represents heterogeneity.

b One SNP associated with hip OA not available in the MI GWAS was removed.

### Causal effect of coronary artery disease and myocardial infarction on osteoarthritis and its phenotypes

After calculating F-statistics and searching the PhenoScanner database, three SNPs associated with confounding factors were removed. Among the rest of the SNPs, three of them were not available in the outcome GWAS. All of the unavailable SNPs were unable to be replaced by their proxy SNPs and were removed. Last, a total of 35 LD-independent genetic variants were taken as IVs for CAD (Supplementary Table 3). Similarly, 20 LD-independent genetic variants were taken as IVs for MI.

As seen in Table 4, using the IVW method, we discovered that CAD was not associated with total OA (OR = 0.96, 95% CI = 0.94, 0.99, *P* = 0.008) and each of its subtypes. The results of the MR-Egger, WM, and MR-PRESSO models were consistent with IVW. Significant heterogeneity was detected by Cochran's *Q*-test for the total, knee, and hip OA (*P* < 0.05). Instead, Cochran's *Q*-test did not present heterogeneity for the spine, hand, and thumb OA. Moreover, the MR-Egger regression result did not reveal any bias by directional pleiotropy.

**Table 4:** MR results of the association between CAD and OA.

**Traits**	**No. of SNPs**	**Method**	**OR (95% CI)**	***P***	**Heterogeneity test**	**Pleiotropy test**
					**Cochran's Q (*P*^a^)**	***P* Intercept**
CAD and Total OA	35	IVW	0.96 (0.94, 0.99)	0.008	66.99 (** <0.001**)	
		WM	0.96 (0.93, 0.99)	0.007		
		MR-Egger	0.93 (0.87, 0.99)	0.035		0.251
		MR-PRESSO (Outlier-corrected)	0.95 (0.93, 0.98)	0.003		
CAD and Knee OA	35	IVW	0.97 (0.92, 1.01)	0.160	73.13 (** <0.001**)	
		WM	0.96 (0.91, 1.00)	0.052		
		MR Egger	0.92 (0.83, 1.02)	0.131		0.301
		MR-PRESSO (Outlier-corrected)	0.96 (0.92, 1.00)	0.085		
CAD and Hip OA	35	IVW	0.96 (0.91, 1.01)	0.085	58.67 (**0.005**)	
		WM	0.96 (0.90, 1.02)	0.177		
		MR Egger	0.90 (0.79, 1.01)	0.090		0.274
		MR-PRESSO (Outlier-corrected)	0.96 (0.92, 1.01)	0.090		
CAD and Spine OA	35	IVW	0.96 (0.91, 1.00)	0.058	38.03 (0.291)	
		WM	0.93 (0.87, 0.99)	0.023		
		MR Egger	0.91 (0.82, 1.02)	0.107		0.351
		MR-PRESSO (Outlier-corrected)	–	–		
CAD and Hand OA	35	IVW	1.01 (0.95, 1.07)	0.804	43.42 (0.129)	
		WM	0.96 (0.87, 1.05)	0.331		
		MR Egger	0.91 (0.79, 1.04)	0.185		0.117
		MR-PRESSO (Outlier-corrected)	–	–		
CAD and Thumb OA	35	IVW	1.00 (0.93, 1.07)	0.937	30.31 (0.649)	
		WM	0.97 (0.86, 1.09)	0.579		
		MR Egger	0.92 (0.77, 1.09)	0.335		0.305
		MR-PRESSO (Outlier-corrected)	–	–		

MR, Mendelian randomization; CAD, coronary artery disease; OA, osteoarthritis; SNPs, single nucleotide polymorphisms; OR, odds ratio; CI, confidence interval; IVW, inverse variance weighted; WM, weighted median; MR-PRESSO, MR-pleiotropy residual sum and outlier.

a Bolded P represents heterogeneity.

As seen in Table 5, we observed that genetically predicted MI was associated with the reduced risk of total OA (IVW OR = 0.95, 95% CI = 0.93, 0.98, *P* = 4E−04) and spine OA (IVW OR = 0.92, 95% CI = 0.88, 0.97, *P* = 0.001) rather than with the knee OA, hip OA, hand OA, and thumb OA after correction for multiple comparisons. Notably, the results of the MR-Egger, WM, and MR-PRESSO models did not reflect a significant association but gave similar effect size estimates (Supplementary Figure 1). No heterogeneity was detected by Cochran's *Q*-test for OA and its phenotypes, except for hip OA (*P* < 0.05). The MR-Egger analysis did not suggest any directional pleiotropy for the IVs. The results of funnel plots and leave-one-out sensitivity analysis demonstrated that no single SNP has a significant effect on the pooled results, verifying the stability of our results, as given in Supplementary Figures 2, 3.

**Table 5:** MR results of the association between MI and OA.

**Traits**	**No. of SNPs**	**Method**	**OR (95% CI)**	***P* ^a^**	**Heterogeneity test**	**Pleiotropy test**
					**Cochran's Q (*P*^b^)**	***P* Intercept**
MI and Total OA	20	IVW	0.95 (0.93, 0.98)	**4E−04**	26.31 (0.122)	
		WM	0.96 (0.93, 0.99)	0.006		
		MR-Egger	0.94 (0.88, 1.00)	0.085		0.699
		MR-PRESSO (Outlier-corrected)	-	-		
MI and Knee OA	20	IVW	0.96 (0.93, 1.00)	0.045	21.23 (0.324)	
		WM	0.96 (0.91, 1.01)	0.140		
		MR Egger	0.95 (0.86, 1.04)	0.245		0.684
		MR-PRESSO (Outlier-corrected)	-	-		
MI and Hip OA	20	IVW	0.95 (0.89, 1.02)	0.145	42.19 (**0.002**)	
		WM	0.93 (0.87, 0.99)	0.028		
		MR Egger	0.88 (0.75, 1.03)	0.121		0.280
		MR-PRESSO (Outlier-corrected)	0.96 (0.90, 1.01)	0.122		
MI and Spine OA	20	IVW	0.92 (0.88, 0.97)	**0.001**	10.59 (0.937)	
		WM	0.91 (0.85, 0.98)	0.014		
		MR Egger	0.96 (0.85, 1.08)	0.525		0.457
		MR-PRESSO (Outlier-corrected)	–	–		
MI and Hand OA	20	IVW	0.98 (0.91, 1.05)	0.531	27.91 (0.085)	
		WM	0.95 (0.86, 1.05)	0.324		
		MR Egger	0.96 (0.80, 1.16)	0.687		0.868
		MR-PRESSO (Outlier-corrected)	–	–		
MI and Thumb OA	20	IVW	1.00 (0.92, 1.09)	0.942	19.15 (0.448)	
		WM	0.98 (0.87, 1.11)	0.768		
		MR Egger	0.94 (0.76, 1.15)	0.540		0.521
		MR-PRESSO (Outlier-corrected)	–	–		

MR, Mendelian randomization; MI, myocardial infarction; OA, osteoarthritis; SNPs, single nucleotide polymorphisms; OR, odds ratio; CI, confidence interval; IVW, inverse variance weighted; WM, weighted median; MR-PRESSO, MR pleiotropy residual sum and outlier.

a Bolded P-value represents statistical significance.

b Bolded P-value represents heterogeneity.

## Discussion

To the best of our knowledge, this is the first two-sample MR study to investigate the bidirectional causal association between OA and CAD as well as MI. The results suggested no causality of OA on CAD and MI. In reverse analyses, genetically increased odds of MI have a causal effect on a lower risk of total and spine OA, while a null association between MI and knee OA, hip OA, hand OA, and thumb OA is provided. No causal effect was discovered for CAD on the risk of OA.

The association of total- and site-specific OA with CAD and MI is still inconclusive. Some cross-sectional studies observed higher CVD risk in patients with OA compared to controls (25, 26). A case–control study indicated that OA was significantly associated with CAD (27). In a cohort study, OA increased the risk of CVD (28). However, a prospective population-based cohort study reported that participants with OA were not at an increased risk of CVD (29). In the present MR study, we revealed a null causality of OA on CAD and MI using primary MR estimate methods (IVW method). Cochran's *Q*-test suggested heterogeneity in the MR (total OA to CAD and MI MR and knee OA to CAD and MI MR). The MR-Egger regression result did not present any bias by directional pleiotropy. However, all of the sensitivity analyses, including the WM, MR-Egger, and MR-PRESSO methods, supported that OA was not causally associated with CAD and MI.

Our findings of a protective relationship between MI and OA are surprising. However, the same association was also observed in observational studies. A cross-sectional study reported an inverse association between CVD and OA after adjustment for age, gender, and CVD risk factors (30). Another case–control study indicated that cardiovascular events were slightly associated with reduced hand OA risk (7). However, these observational associations cannot provide adequate evidence of causality since they are influenced by confounding factors and reverse causality. Our MR study explored the causality from the association of a selected exposure, predicted by genetic variants, with corresponding outcomes, which can avoid these biases. The MR result is in favor of the causal association between MI and total (*P* = 4E−04) and spine OA (*P* = 0.001), using the IVW method. Potential mechanisms for a protective effect of MI on OA involve inhibition of autophagy and inhibition of inflammation. After MI, cardiomyocytes appear as oxidative stress and autophagic cell death under the mediation of myocardin and p53. To inhibit the induction of autophagy, long non-coding RNA (lncRNA) cardiac autophagy inhibitory factor (CAIF) was correspondingly overexpressed (31). Meanwhile, CAIF overexpression mediates the downregulation of miR-1246 and inhibits the secretion of interleukin-6 (IL-6) from CHON-001 cells. Therefore, CAIF would inhibit inflammation through the CAIF/miR-1246/IL-6 pathway to improve OA (32). However, the inhibition of autophagy and the inhibition of inflammation may be the result of disease compensation, and, therefore, may be related to disease progression rather than disease initiation. A further longitudinal study of the MI progression rate in OA coexisting with MI may confirm this notion. We did not find causal associations between genetically predicted MI and the knee, hip, hand, or thumb OA, revealing the joint site's specificity. The site specificity of the causal relationship may be caused by the association of spondyloarthropathy with aortitis and biomechanical differences.

This study has some advantages. First, our study is the first MR study to identify the bidirectional causal relationship between multiple OA phenotypes and CAD, as well as MI, in the largest OA GWAS to date. Second, the MR method is used to infer the causal relationship between two diseases. This is not feasible in randomized controlled trials owing to an ethical problem to let patients with one disease not receive treatment to observe the development of another disease. Third, using summary data from the GWAS dataset in two-sample MR increase statistical power, especially in testing the causality of binary disease outcomes (33). Furthermore, given the possible genetic interaction between CVD and OA, we performed bidirectional MR analysis and observed negative evidence in only one direction.

However, this study has several limitations. First, with summary data from a large GWAS dataset, it is unlikely to be able to perform an analysis on the relevant stratifying risk factors, such as the duration of the disease, severity and treatment undertaken, and comorbidities. An independent cohort with individual-level data is warranted to replicate the findings later. Besides, participants with CAD and MI were not screened for OA at baseline and vice versa. Many exposed datasets with outcomes would lead to exaggerated causal estimates in the MR analysis. However, this is an inevitable limitation of two-sample MR analysis in the absence of individual-level data. Finally, not all the study participants are of European ancestry, and the variability of allele frequencies between populations may affect the results.

In conclusion, this MR study does not observe the causal effect of OA on CAD and MI or the causal effect of CAD on OA. Notably, we found a causal association between MI and total and spine OA but a null association between MI and knee OA, hip OA, hand OA, and thumb OA.

## Data availability statement

The original contributions presented in the study are included in the article/Supplementary material, further inquiries can be directed to the corresponding author/s.

## Ethics statement

Ethical review and approval was not required for this study in accordance with the local legislation and institutional requirements.

## Author contributions

HX mainly designed and performed analysis, verified data, and wrote the manuscript. YLin, HJ, and YLi performed experiments and analysis. MJ supervised the entire project. All authors have read and approved the final manuscript and provided critical feedback on intellectual content.

## Conflict of interest

The authors declare that the research was conducted in the absence of any commercial or financial relationships that could be construed as a potential conflict of interest.

## Publisher's note

All claims expressed in this article are solely those of the authors and do not necessarily represent those of their affiliated organizations, or those of the publisher, the editors and the reviewers. Any product that may be evaluated in this article, or claim that may be made by its manufacturer, is not guaranteed or endorsed by the publisher.
