# Supplementary material for: Osteoarthritis, coronary artery disease, and myocardial infarction: A mendelian randomization study
Source: Front Cardiovasc Med. 2022 Aug 25;9:892742. doi: 10.3389/fcvm.2022.892742 (PMC9452687; doi:10.3389/fcvm.2022.892742)

| Supplementary Table S1 Characteristics of SNPs used as genetic instruments for OA and its other phenotypes in the present MR study | | | | | | | | | | | | | | |
| --- | --- | --- | --- | --- | --- | --- | --- | --- | --- | --- | --- | --- | --- | --- |
| Exposure | SNP | Chr | Position | EA | NEA | EAF | SNP-Exposure association | | | *R^2^* ^a^ | *F*-statistic ^b^ | Proxy ^c^ | Remove ^d^ | Confounder ^e^ |
|  |  |  |  |  |  |  | Beta | SE | *P* value |  |  |  |  |  |
| Total OA | rs2622873 | 1 | 103466053 | T | C | 0.88 | 0.046 | 0.007 | 4.24E-11 | 0.0001 | 43 |  |  |  |
| Total OA | chr1:150214028 | 1 | 150214028 | D | I | 0.38 | 0.036 | 0.006 | 8.58E-10 | 0.0000 | 36 |  | r^2^ > 0.01 |  |
| Total OA | rs62182810 | 2 | 204387482 | A | G | 0.54 | 0.027 | 0.005 | 3.82E-09 | 0.0000 | 29 |  |  |  |
| Total OA | rs3771501 | 2 | 70717653 | A | G | 0.47 | 0.036 | 0.005 | 4.05E-15 | 0.0001 | 52 |  |  |  |
| Total OA | rs62242105 | 3 | 20630395 | G | A | 0.67 | 0.029 | 0.005 | 2.93E-09 | 0.0000 | 35 |  |  |  |
| Total OA | rs11729628 | 4 | 121592875 | G | T | 0.76 | 0.031 | 0.005 | 4.74E-09 | 0.0000 | 38 |  |  |  |
| Total OA | rs11731421 | 4 | 1750487 | A | G | 0.35 | 0.031 | 0.005 | 1.88E-10 | 0.0000 | 38 |  |  |  |
| Total OA | rs13107325 | 4 | 103188709 | T | C | 0.07 | 0.078 | 0.009 | 3.25E-17 | 0.0001 | 75 |  |  | Body mass index |
| Total OA | rs201194999 | 4 | 66666895 | C | T | 0.70 | 0.126 | 0.021 | 3.05E-09 | 0.0000 | 35 |  | r^2^ > 0.01 |  |
| Total OA | rs1913707 | 4 | 13043758 | A | G | 0.61 | 0.033 | 0.005 | 1.39E-12 | 0.0001 | 44 |  |  |  |
| Total OA | rs17677555 | 5 | 127852612 | C | G | 0.25 | 0.030 | 0.005 | 1.10E-08 | 0.0000 | 36 |  |  |  |
| Total OA | rs12667224 | 7 | 114024316 | G | A | 0.48 | 0.027 | 0.005 | 1.66E-09 | 0.0000 | 29 |  |  |  |
| Total OA | rs4979341 | 9 | 116905543 | T | C | 0.27 | 0.032 | 0.005 | 1.39E-09 | 0.0000 | 41 |  |  |  |
| Total OA | rs10831476 | 11 | 95796910 | A | C | 0.81 | 0.033 | 0.006 | 7.77E-09 | 0.0000 | 30 |  |  |  |
| Total OA | rs12901372 | 15 | 67370506 | C | G | 0.53 | 0.030 | 0.005 | 1.02E-10 | 0.0000 | 36 |  |  |  |
| Total OA | rs1401795 | 17 | 54839652 | A | G | 0.50 | 0.027 | 0.005 | 6.19E-09 | 0.0000 | 29 |  |  |  |
| Total OA | rs216175 | 17 | 2167690 | A | C | 0.83 | 0.042 | 0.006 | 2.74E-12 | 0.0001 | 49 |  |  |  |
| Total OA | rs1039257158 | 18 | 77950448 | T | C | 0.00 | 1.287 | 0.222 | 6.56E-09 | 0.0000 | 34 |  | r^2^ > 0.01 |  |
| Total OA | rs10405617 | 19 | 10752968 | A | G | 0.32 | 0.032 | 0.005 | 9.33E-11 | 0.0000 | 41 |  |  |  |
| Total OA | rs75621460 | 19 | 41833784 | A | G | 0.03 | 0.096 | 0.016 | 1.06E-09 | 0.0000 | 36 |  |  |  |
| Total OA | rs2425061 | 20 | 33968067 | A | G | 0.63 | 0.033 | 0.005 | 2.14E-12 | 0.0001 | 44 |  |  | Waist hip ratio |
| Knee OA | rs2791549 | 1 | 219658003 | A | C | 0.30 | 0.046 | 0.008 | 3.06E-09 | 0.0001 | 33 |  |  |  |
| Knee OA | rs72979233 | 11 | 74355523 | G | A | 0.24 | 0.050 | 0.008 | 2.54E-09 | 0.0001 | 39 |  |  |  |
| Knee OA | rs1426371 | 12 | 108629780 | G | A | 0.73 | 0.052 | 0.008 | 8.86E-10 | 0.0001 | 42 |  |  |  |
| Knee OA | rs753350451 | 12 | 123732769 | I | D | 0.80 | 0.070 | 0.011 | 3.36E-10 | 0.0001 | 40 |  | r^2^ > 0.01 |  |
| Knee OA | rs10842226 | 12 | 23959589 | A | G | 0.42 | 0.047 | 0.008 | 3.63E-09 | 0.0001 | 35 |  |  |  |
| Knee OA | rs7967762 | 12 | 48420214 | T | C | 0.17 | 0.057 | 0.010 | 2.09E-09 | 0.0001 | 32 |  |  |  |
| Knee OA | rs58973023 | 13 | 42959133 | A | T | 0.49 | 0.056 | 0.009 | 4.72E-10 | 0.0001 | 39 |  |  |  |
| Knee OA | rs4380013 | 15 | 50759428 | A | G | 0.19 | 0.056 | 0.009 | 8.73E-10 | 0.0001 | 39 |  |  |  |
| Knee OA | rs4775006 | 15 | 58215727 | A | C | 0.42 | 0.046 | 0.008 | 8.55E-10 | 0.0001 | 33 |  |  |  |
| Knee OA | rs12914479 | 15 | 99174828 | C | G | 0.66 | 0.044 | 0.008 | 7.12E-09 | 0.0001 | 30 |  |  |  |
| Knee OA | rs6500609 | 16 | 4515334 | G | C | 0.89 | 0.067 | 0.011 | 5.16E-09 | 0.0001 | 37 |  |  |  |
| Knee OA | rs9940278 | 16 | 53800200 | T | C | 0.44 | 0.058 | 0.007 | 3.19E-16 | 0.0002 | 69 |  |  | Body mass index |
| Knee OA | rs34195470 | 16 | 69955690 | G | A | 0.55 | 0.052 | 0.007 | 3.13E-13 | 0.0001 | 55 |  |  |  |
| Knee OA | rs4548913 | 17 | 2209888 | G | A | 0.37 | 0.051 | 0.007 | 3.15E-12 | 0.0001 | 53 |  |  |  |
| Knee OA | rs2163832 | 19 | 10745764 | T | C | 0.32 | 0.045 | 0.008 | 2.73E-09 | 0.0001 | 32 |  | r^2^ > 0.01 |  |
| Knee OA | rs7581446 | 2 | 33423801 | C | T | 0.52 | 0.057 | 0.009 | 1.71E-10 | 0.0001 | 40 |  |  |  |
| Knee OA | rs66906321 | 2 | 630070 | C | T | 0.82 | 0.057 | 0.009 | 1.71E-09 | 0.0001 | 40 |  |  | Body mass index |
| Knee OA | rs143384 | 20 | 34025756 | A | G | 0.59 | 0.072 | 0.007 | 1.01E-23 | 0.0003 | 106 |  |  | Waist hip ratio |
| Knee OA | rs11705555 | 22 | 28206912 | A | C | 0.76 | 0.051 | 0.009 | 2.99E-09 | 0.0001 | 32 |  |  |  |
| Knee OA | rs7680647 | 4 | 1750487 | C | T | 0.37 | 0.044 | 0.008 | 1.24E-08 | 0.0001 | 30 |  |  |  |
| Knee OA | rs10038860 | 5 | 141809938 | A | G | 0.27 | 0.046 | 0.008 | 5.62E-09 | 0.0001 | 33 |  |  |  |
| Knee OA | rs2066928 | 5 | 30843787 | G | A | 0.52 | 0.041 | 0.007 | 1.20E-08 | 0.0001 | 34 | Not available |  |  |
| Knee OA | rs72760655 | 9 | 116916214 | A | C | 0.33 | 0.049 | 0.008 | 7.25E-11 | 0.0001 | 38 |  |  |  |
| Knee OA | rs10974438 | 9 | 4291928 | A | C | 0.64 | 0.043 | 0.007 | 4.89E-09 | 0.0001 | 38 |  |  |  |
| Hip OA | rs11164653 | 1 | 103464210 | C | T | 0.59 | 0.080 | 0.009 | 2.77E-18 | 0.0002 | 79 |  |  |  |
| Hip OA | rs4411121 | 1 | 118757034 | T | C | 0.31 | 0.065 | 0.010 | 2.16E-11 | 0.0001 | 42 |  |  |  |
| Hip OA | rs1046934 | 1 | 184023529 | A | C | 0.65 | 0.071 | 0.009 | 3.81E-14 | 0.0002 | 62 |  |  |  |
| Hip OA | rs2605098 | 1 | 219643649 | A | G | 0.34 | 0.065 | 0.010 | 6.83E-12 | 0.0001 | 42 |  |  | Waist hip ratio |
| Hip OA | rs3740129 | 10 | 73767859 | A | G | 0.45 | 0.055 | 0.009 | 1.84E-09 | 0.0001 | 37 |  |  |  |
| Hip OA | rs67924081 | 11 | 65342981 | A | G | 0.74 | 0.064 | 0.010 | 7.80E-10 | 0.0001 | 41 |  |  |  |
| Hip OA | rs34560402 | 11 | 66872320 | C | T | 0.94 | 0.119 | 0.020 | 1.59E-09 | 0.0001 | 35 |  | r^2^ > 0.01 |  |
| Hip OA | rs10831477 | 11 | 95797111 | T | G | 0.81 | 0.070 | 0.012 | 1.20E-09 | 0.0001 | 34 |  |  |  |
| Hip OA | rs1809889 | 12 | 124801226 | T | C | 0.28 | 0.060 | 0.010 | 3.58E-09 | 0.0001 | 36 |  |  |  |
| Hip OA | rs10843013 | 12 | 28025196 | C | A | 0.22 | 0.111 | 0.011 | 2.91E-24 | 0.0003 | 102 |  |  |  |
| Hip OA | rs79056043 | 12 | 59289598 | G | A | 0.06 | 0.114 | 0.019 | 1.99E-09 | 0.0001 | 36 |  |  |  |
| Hip OA | rs746239049 | 15 | 63067433 | I | D | 0.79 | 0.085 | 0.014 | 3.35E-10 | 0.0001 | 37 |  | r^2^ > 0.01 |  |
| Hip OA | rs12908498 | 15 | 67366488 | C | G | 0.54 | 0.080 | 0.010 | 1.85E-16 | 0.0002 | 64 |  |  |  |
| Hip OA | rs9940278 | 16 | 53800200 | T | C | 0.44 | 0.054 | 0.009 | 1.77E-09 | 0.0001 | 36 |  |  | Body mass index |
| Hip OA | rs1401796 | 17 | 54839759 | C | A | 0.49 | 0.058 | 0.009 | 1.43E-10 | 0.0001 | 42 |  |  |  |
| Hip OA | rs7222178 | 17 | 59652282 | A | T | 0.19 | 0.067 | 0.012 | 7.35E-09 | 0.0001 | 31 |  |  |  |
| Hip OA | rs2521348 | 17 | 67499717 | T | C | 0.39 | 0.056 | 0.009 | 1.56E-09 | 0.0001 | 39 |  |  |  |
| Hip OA | rs4252548 | 19 | 55879672 | T | C | 0.03 | 0.225 | 0.028 | 2.24E-15 | 0.0002 | 65 |  |  |  |
| Hip OA | rs66989638 | 2 | 106689736 | A | G | 0.13 | 0.079 | 0.014 | 4.79E-09 | 0.0001 | 32 |  |  |  |
| Hip OA | rs2862851 | 2 | 70712802 | T | C | 0.47 | 0.066 | 0.009 | 3.86E-13 | 0.0002 | 54 |  |  |  |
| Hip OA | rs12160491 | 22 | 38195796 | G | A | 0.30 | 0.061 | 0.010 | 4.37E-10 | 0.0001 | 37 |  |  |  |
| Hip OA | rs781661531 | 3 | 187051013 | C | T | 0.00 | 2.254 | 0.347 | 8.36E-11 | 0.0001 | 42 |  | r^2^ > 0.01 |  |
| Hip OA | rs747952496 | 3 | 188311659 | A | G | 0.00 | 1.949 | 0.296 | 4.91E-11 | 0.0001 | 43 |  | r^2^ > 0.01 |  |
| Hip OA | rs9835230 | 3 | 189735461 | A | G | 0.24 | 0.064 | 0.011 | 1.34E-09 | 0.0001 | 34 |  |  |  |
| Hip OA | rs2268023 | 3 | 52819327 | A | T | 0.41 | 0.068 | 0.009 | 1.56E-13 | 0.0002 | 57 |  |  | Body mass index |
| Hip OA | rs6855246 | 4 | 103112470 | G | A | 0.07 | 0.109 | 0.019 | 7.94E-09 | 0.0001 | 33 |  |  |  |
| Hip OA | rs1913707 | 4 | 13039440 | A | G | 0.60 | 0.068 | 0.009 | 1.82E-13 | 0.0002 | 57 |  |  |  |
| Hip OA | rs798756 | 4 | 1707447 | C | T | 0.81 | 0.068 | 0.011 | 2.24E-09 | 0.0001 | 38 |  |  |  |
| Hip OA | rs17677724 | 5 | 128015370 | T | C | 0.16 | 0.072 | 0.012 | 3.54E-09 | 0.0001 | 36 |  |  |  |
| Hip OA | rs4073717 | 5 | 170864021 | G | T | 0.80 | 0.067 | 0.011 | 2.54E-09 | 0.0001 | 37 |  |  |  |
| Hip OA | rs10940168 | 5 | 67823586 | G | A | 0.61 | 0.053 | 0.009 | 7.74E-09 | 0.0001 | 35 |  |  |  |
| Hip OA | rs79220007 | 6 | 26098474 | C | T | 0.07 | 0.106 | 0.018 | 2.22E-09 | 0.0001 | 35 |  |  |  |
| Hip OA | rs6908606 | 6 | 44998888 | G | A | 0.29 | 0.069 | 0.010 | 3.86E-12 | 0.0001 | 48 |  |  |  |
| Hip OA | rs9475400 | 6 | 55638258 | T | C | 0.10 | 0.108 | 0.015 | 8.03E-13 | 0.0001 | 52 |  |  |  |
| Hip OA | rs12209223 | 6 | 76164589 | A | C | 0.11 | 0.140 | 0.014 | 1.88E-22 | 0.0003 | 100 |  |  |  |
| Hip OA | rs143083812 | 7 | 128843410 | T | C | 0.00 | 1.063 | 0.164 | 8.21E-11 | 0.0001 | 42 |  | r^2^ > 0.01 |  |
| Hip OA | rs111844273 | 7 | 18436337 | A | G | 0.02 | 0.232 | 0.033 | 1.05E-12 | 0.0001 | 49 |  |  |  |
| Hip OA | rs765002298 | 8 | 130733847 | I | D | 0.80 | 0.106 | 0.013 | 1.75E-15 | 0.0002 | 66 |  | r^2^ > 0.01 |  |
| Hip OA | rs79895530 | 9 | 110416422 | C | T | 0.87 | 0.101 | 0.013 | 7.04E-14 | 0.0001 | 60 |  |  |  |
| Hip OA | rs1330349 | 9 | 117840742 | C | G | 0.59 | 0.063 | 0.009 | 6.94E-12 | 0.0001 | 49 |  |  |  |
| Hip OA | rs7862601 | 9 | 118343026 | G | A | 0.38 | 0.063 | 0.011 | 6.19E-09 | 0.0001 | 33 |  |  |  |
| Hip OA | rs2416564 | 9 | 119370679 | C | T | 0.40 | 0.073 | 0.009 | 1.00E-15 | 0.0002 | 66 |  |  |  |
| Hip OA | rs10983775 | 9 | 120521100 | C | T | 0.46 | 0.053 | 0.009 | 4.65E-09 | 0.0001 | 35 |  |  |  |
| Hip OA | rs12377624 | 9 | 129373110 | G | C | 0.64 | 0.063 | 0.010 | 4.59E-11 | 0.0001 | 40 |  |  |  |
| Hip OA | rs10465114 | 9 | 129917824 | A | G | 0.22 | 0.063 | 0.011 | 9.04E-09 | 0.0001 | 33 |  |  |  |
| Spine OA | rs201194999 | 4 | 66666895 | C | T | 0.69 | 0.167 | 0.029 | 1.24E-08 | 0.0001 | 33 | Not available |  |  |
| Hand OA | rs10062749 | 5 | 141805088 | T | G | 0.27 | 0.081 | 0.014 | 2.04E-09 | 0.0001 | 33 |  |  |  |
| Hand OA | rs11071366 | 15 | 58344290 | T | A | 0.39 | 0.104 | 0.012 | 4.88E-17 | 0.0002 | 75 |  |  |  |
| Hand OA | rs1560080 | 5 | 115338732 | G | A | 0.17 | 0.092 | 0.016 | 9.61E-09 | 0.0001 | 33 |  |  |  |
| Hand OA | rs3771498 | 2 | 70720070 | C | T | 0.48 | 0.079 | 0.012 | 6.81E-11 | 0.0001 | 43 |  |  |  |
| Hand OA | rs3993110 | 11 | 12809152 | A | C | 0.61 | 0.082 | 0.012 | 3.75E-11 | 0.0002 | 47 |  |  |  |
| Hand OA | rs7748189 | 6 | 18385889 | A | G | 0.73 | 0.080 | 0.014 | 6.13E-09 | 0.0001 | 33 |  |  |  |
| Hand OA | rs8112559 | 19 | 46390455 | C | G | 0.89 | 0.126 | 0.019 | 7.32E-11 | 0.0001 | 44 |  |  |  |
| Thumb OA | rs10062749 | 5 | 141805088 | T | G | 0.27 | 0.106 | 0.019 | 1.29E-08 | 0.0001 | 31 |  |  |  |
| Thumb OA | rs2862851 | 2 | 70712802 | T | C | 0.46 | 0.105 | 0.017 | 3.24E-10 | 0.0002 | 38 |  |  |  |
| Thumb OA | rs4238326 | 15 | 58336000 | C | T | 0.39 | 0.117 | 0.017 | 7.30E-12 | 0.0002 | 47 |  |  |  |
| Thumb OA | rs11588850 | 1 | 227927242 | A | G | 0.18 | 0.134 | 0.021 | 3.53E-10 | 0.0002 | 41 |  |  |  |
| Abbreviation: SNP, single nucleotide polymorphism; Chr, chromosome; EA, effect allele; NEA, non-effect allele; EAF, effect allele frequency; SE, standard error; OA, osteoarthritis.  ^a^ *R^2^* was calculated using the following formula: (2×EAF×(1-EAF)×beta^2^)/[(2×EAF×(1-EAF)×beta^2^)+(2×EAF×(1-EAF)×N×SE^2^)], where EAF is the effect allele frequency, beta is the estimated effect on exposure, Ν is the sample size of the GWAS for the SNP-exposure association and SE is the standard error of the estimated effect.  ^b^ *F* statistic was calculated using the following formula: *R^2^*(N-2)/(1-*R^2^*), where *R^2^* is the proportion of variance in exposure explained by each instrument and N is the sample size of the GWAS for the SNP-exposure association.  ^c^ SNPs which were not available in the outcome GWAS were replaced by their proxy (r^2^ >0.8) or deleted if the proxy was also not available.  ^d^ SNPs with r^2^ > 0.01 were removed.  ^e^ SNPs associated with confounding factors were removed after searching Phenoscanner database. | | | | | | | | | | | | | | |

| Supplementary Table S2 Genetic association of genetic variants with CAD and MI | | | | | | | | | | |
| --- | --- | --- | --- | --- | --- | --- | --- | --- | --- | --- |
| Exposure | SNP | EA | NEA | CAD | | |  | MI | | |
|  |  |  |  | Beta | SE | *P* value |  | Beta | SE | *P* value |
| Total OA | rs2622873 | T | C | 0.010 | 0.014 | 0.47 |  | 0.019 | 0.016 | 0.24 |
| Total OA | rs62182810 | A | G | -0.047 | 0.010 | 0.00 |  | -0.038 | 0.010 | 0.00 |
| Total OA | rs3771501 | A | G | 0.008 | 0.010 | 0.39 |  | 0.003 | 0.010 | 0.78 |
| Total OA | rs62242105 | G | A | -0.020 | 0.011 | 0.06 |  | -0.028 | 0.012 | 0.02 |
| Total OA | rs11729628 | G | T | -0.008 | 0.013 | 0.52 |  | 0.002 | 0.014 | 0.90 |
| Total OA | rs11731421 | A | G | -0.003 | 0.010 | 0.76 |  | -0.007 | 0.011 | 0.54 |
| Total OA | rs1913707 | A | G | -0.005 | 0.009 | 0.56 |  | -0.002 | 0.010 | 0.88 |
| Total OA | rs17677555 | C | G | 0.017 | 0.011 | 0.14 |  | 0.006 | 0.012 | 0.61 |
| Total OA | rs12667224 | G | A | -0.016 | 0.009 | 0.09 |  | -0.010 | 0.010 | 0.35 |
| Total OA | rs4979341 | T | C | 0.000 | 0.011 | 0.99 |  | -0.008 | 0.012 | 0.48 |
| Total OA | rs10831476 | A | C | -0.017 | 0.012 | 0.17 |  | -0.017 | 0.013 | 0.20 |
| Total OA | rs12901372 | C | G | 0.004 | 0.010 | 0.68 |  | 0.010 | 0.011 | 0.35 |
| Total OA | rs1401795 | A | G | -0.005 | 0.010 | 0.59 |  | -0.005 | 0.011 | 0.60 |
| Total OA | rs216175 | A | C | 0.030 | 0.012 | 0.01 |  | 0.033 | 0.013 | 0.01 |
| Total OA | rs10405617 | A | G | -0.043 | 0.010 | 0.00 |  | -0.037 | 0.011 | 0.00 |
| Total OA | rs75621460 | A | G | -0.127 | 0.045 | 0.00 |  | -0.069 | 0.049 | 0.16 |
| Knee OA | rs2791549 | A | C | -0.010 | 0.010 | 0.32 |  | -0.005 | 0.012 | 0.66 |
| Knee OA | rs72979233 | G | A | 0.009 | 0.011 | 0.41 |  | 0.014 | 0.012 | 0.24 |
| Knee OA | rs1426371 | G | A | -0.006 | 0.011 | 0.58 |  | -0.003 | 0.012 | 0.80 |
| Knee OA | rs10842226 | A | G | -0.002 | 0.010 | 0.86 |  | -0.001 | 0.011 | 0.91 |
| Knee OA | rs7967762 | T | C | 0.007 | 0.011 | 0.53 |  | 0.023 | 0.013 | 0.08 |
| Knee OA | rs58973023 | A | T | -0.005 | 0.010 | 0.61 |  | -0.004 | 0.011 | 0.68 |
| Knee OA | rs4380013 | A | G | 0.017 | 0.012 | 0.14 |  | 0.010 | 0.013 | 0.45 |
| Knee OA | rs4775006 | A | C | -0.023 | 0.010 | 0.02 |  | -0.011 | 0.011 | 0.29 |
| Knee OA | rs12914479 | C | G | 0.003 | 0.010 | 0.73 |  | 0.002 | 0.011 | 0.84 |
| Knee OA | rs6500609 | G | C | 0.020 | 0.015 | 0.19 |  | 0.037 | 0.017 | 0.03 |
| Knee OA | rs34195470 | G | A | -0.007 | 0.010 | 0.49 |  | 0.001 | 0.011 | 0.95 |
| Knee OA | rs4548913 | G | A | -0.037 | 0.010 | 0.00 |  | -0.040 | 0.011 | 0.00 |
| Knee OA | rs7581446 | C | T | -0.010 | 0.009 | 0.31 |  | -0.008 | 0.010 | 0.43 |
| Knee OA | rs11705555 | A | C | -0.022 | 0.012 | 0.07 |  | -0.029 | 0.013 | 0.03 |
| Knee OA | rs7680647 | C | T | 0.003 | 0.010 | 0.78 |  | 0.005 | 0.011 | 0.68 |
| Knee OA | rs10038860 | A | G | 0.002 | 0.011 | 0.87 |  | -0.002 | 0.012 | 0.84 |
| Knee OA | rs72760655 | A | C | -0.006 | 0.010 | 0.53 |  | -0.006 | 0.011 | 0.59 |
| Knee OA | rs10974438 | A | C | 0.000 | 0.010 | 0.99 |  | 0.005 | 0.011 | 0.67 |
| Hip OA | rs11164653 | C | T | -0.015 | 0.010 | 0.13 |  | -0.016 | 0.011 | 0.14 |
| Hip OA | rs4411121 | T | C | -0.020 | 0.010 | 0.04 |  | -0.009 | 0.011 | 0.39 |
| Hip OA | rs1046934 | A | C | -0.028 | 0.010 | 0.00 |  | -0.024 | 0.011 | 0.03 |
| Hip OA | rs3740129 | A | G | 0.006 | 0.010 | 0.56 |  | 0.006 | 0.011 | 0.59 |
| Hip OA | rs67924081 | A | G | 0.011 | 0.011 | 0.34 |  | 0.010 | 0.012 | 0.42 |
| Hip OA | rs10831477 | T | G | -0.016 | 0.012 | 0.17 |  | -0.017 | 0.013 | 0.21 |
| Hip OA | rs1809889 | T | C | 0.005 | 0.010 | 0.60 |  | 0.005 | 0.012 | 0.66 |
| Hip OA | rs10843013 | C | A | 0.009 | 0.012 | 0.44 |  | 0.010 | 0.013 | 0.46 |
| Hip OA | rs79056043 | G | A | -0.026 | 0.019 | 0.18 |  | -0.037 | 0.022 | 0.09 |
| Hip OA | rs12908498 | C | G | 0.003 | 0.009 | 0.72 |  | 0.004 | 0.010 | 0.67 |
| Hip OA | rs1401796 | C | A | 0.001 | 0.010 | 0.92 |  | 0.008 | 0.010 | 0.45 |
| Hip OA | rs7222178 | A | T | -0.003 | 0.012 | 0.77 |  | 0.004 | 0.013 | 0.73 |
| Hip OA | rs2521348 | T | C | 0.009 | 0.009 | 0.32 |  | 0.002 | 0.010 | 0.87 |
| Hip OA | rs4252548 | T | C | -0.019 | 0.046 | 0.69 |  | NA | NA | NA |
| Hip OA | rs66989638 | A | G | -0.005 | 0.016 | 0.74 |  | 0.008 | 0.018 | 0.67 |
| Hip OA | rs2862851 | T | C | 0.009 | 0.009 | 0.36 |  | 0.003 | 0.010 | 0.79 |
| Hip OA | rs12160491 | G | A | -0.011 | 0.010 | 0.29 |  | -0.017 | 0.011 | 0.12 |
| Hip OA | rs9835230 | A | G | 0.018 | 0.011 | 0.10 |  | 0.009 | 0.012 | 0.45 |
| Hip OA | rs6855246 | G | A | -0.001 | 0.024 | 0.95 |  | -0.004 | 0.026 | 0.89 |
| Hip OA | rs1913707 | A | G | -0.005 | 0.009 | 0.56 |  | -0.002 | 0.010 | 0.88 |
| Hip OA | rs798756 | C | T | 0.028 | 0.012 | 0.02 |  | 0.037 | 0.013 | 0.00 |
| Hip OA | rs17677724 | T | C | 0.026 | 0.013 | 0.05 |  | 0.017 | 0.015 | 0.24 |
| Hip OA | rs4073717 | G | T | 0.010 | 0.012 | 0.37 |  | -0.001 | 0.013 | 0.91 |
| Hip OA | rs10940168 | G | A | 0.000 | 0.010 | 0.97 |  | 0.010 | 0.011 | 0.37 |
| Hip OA | rs79220007 | C | T | 0.039 | 0.024 | 0.11 |  | 0.042 | 0.027 | 0.12 |
| Hip OA | rs6908606 | G | A | 0.008 | 0.011 | 0.45 |  | 0.001 | 0.012 | 0.93 |
| Hip OA | rs9475400 | T | C | -0.006 | 0.014 | 0.66 |  | -0.025 | 0.016 | 0.11 |
| Hip OA | rs12209223 | A | C | 0.024 | 0.016 | 0.13 |  | 0.008 | 0.018 | 0.65 |
| Hip OA | rs111844273 | A | G | -0.009 | 0.040 | 0.82 |  | -0.001 | 0.043 | 0.98 |
| Hip OA | rs79895530 | C | T | -0.009 | 0.013 | 0.49 |  | 0.004 | 0.015 | 0.77 |
| Hip OA | rs1330349 | C | G | -0.006 | 0.009 | 0.55 |  | -0.002 | 0.010 | 0.87 |
| Hip OA | rs7862601 | G | A | -0.004 | 0.009 | 0.71 |  | 0.003 | 0.011 | 0.75 |
| Hip OA | rs2416564 | C | T | -0.002 | 0.010 | 0.81 |  | 0.001 | 0.011 | 0.93 |
| Hip OA | rs10983775 | C | T | -0.011 | 0.009 | 0.25 |  | -0.008 | 0.010 | 0.42 |
| Hip OA | rs12377624 | G | C | -0.007 | 0.010 | 0.48 |  | 0.008 | 0.011 | 0.50 |
| Hip OA | rs10465114 | A | G | -0.001 | 0.011 | 0.94 |  | -0.004 | 0.012 | 0.72 |
| Hand OA | rs10062749 | T | G | 0.003 | 0.011 | 0.78 |  | -0.001 | 0.012 | 0.96 |
| Hand OA | rs11071366 | T | A | -0.024 | 0.010 | 0.02 |  | -0.013 | 0.011 | 0.24 |
| Hand OA | rs1560080 | G | A | -0.004 | 0.012 | 0.74 |  | -0.006 | 0.013 | 0.66 |
| Hand OA | rs3771498 | C | T | -0.005 | 0.009 | 0.58 |  | 0.000 | 0.010 | 1.00 |
| Hand OA | rs3993110 | A | C | -0.019 | 0.009 | 0.04 |  | -0.019 | 0.011 | 0.07 |
| Hand OA | rs7748189 | A | G | 0.018 | 0.010 | 0.08 |  | 0.013 | 0.011 | 0.24 |
| Hand OA | rs8112559 | C | G | 0.000 | 0.016 | 0.99 |  | 0.009 | 0.017 | 0.62 |
| Thumb OA | rs10062749 | T | G | 0.003 | 0.011 | 0.78 |  | -0.001 | 0.012 | 0.96 |
| Thumb OA | rs2862851 | T | C | 0.009 | 0.009 | 0.36 |  | 0.003 | 0.010 | 0.79 |
| Thumb OA | rs4238326 | C | T | -0.023 | 0.010 | 0.02 |  | -0.010 | 0.012 | 0.38 |
| Thumb OA | rs11588850 | A | G | 0.006 | 0.013 | 0.64 |  | 0.016 | 0.014 | 0.25 |
| Abbreviation: CAD, coronary artery disease; MI, myocardial infarction; SNP, single nucleotide polymorphism; EA, effect allele; NEA, non-effect allele; SE, standard error; OA, osteoarthritis. | | | | | | | | | | |

| Supplementary Table S3 Characteristics of SNPs used as genetic instruments for CAD and MI in the present MR study | | | | | | | | | | | | | | |
| --- | --- | --- | --- | --- | --- | --- | --- | --- | --- | --- | --- | --- | --- | --- |
| Exposure | SNP | Chr | Position | EA | NEA | EAF | SNP-Exposure association | | | *R^2^* ^a^ | *F*-statistic ^b^ | Proxy ^c^ | Remove ^d^ | Confounder ^e^ |
|  |  |  |  |  |  |  | Beta | SE | *P* value |  |  |  |  |  |
| CAD | rs7528419 | 1 | 109817192 | A | G | 0.79 | 0.113 | 0.011 | 1.97E-23 | 0.0006 | 106 |  |  |  |
| CAD | rs6689306 | 1 | 154395946 | A | G | 0.45 | 0.058 | 0.010 | 2.60E-09 | 0.0002 | 34 |  |  |  |
| CAD | rs67180937 | 1 | 222823743 | G | T | 0.66 | 0.077 | 0.012 | 1.01E-12 | 0.0002 | 41 |  |  |  |
| CAD | rs11206510 | 1 | 55496039 | T | C | 0.85 | 0.077 | 0.014 | 2.34E-08 | 0.0002 | 30 |  |  |  |
| CAD | rs9970807 | 1 | 56965664 | C | T | 0.92 | 0.122 | 0.016 | 5.00E-14 | 0.0003 | 58 |  |  |  |
| CAD | rs11191416 | 10 | 104604916 | T | G | 0.87 | 0.077 | 0.014 | 4.65E-09 | 0.0002 | 30 |  |  | Body mass index |
| CAD | rs2487928 | 10 | 30323892 | A | G | 0.42 | 0.058 | 0.010 | 4.41E-11 | 0.0002 | 34 |  |  |  |
| CAD | rs1870634 | 10 | 44480811 | G | T | 0.64 | 0.077 | 0.009 | 5.55E-15 | 0.0004 | 73 |  |  |  |
| CAD | rs1412444 | 10 | 91002927 | T | C | 0.37 | 0.068 | 0.010 | 5.15E-12 | 0.0003 | 46 |  |  |  |
| CAD | rs2128739 | 11 | 103673277 | A | C | 0.32 | 0.068 | 0.010 | 7.05E-11 | 0.0003 | 46 |  |  |  |
| CAD | rs10840293 | 11 | 9751196 | A | G | 0.55 | 0.058 | 0.010 | 1.28E-08 | 0.0002 | 34 |  |  |  |
| CAD | rs11065979 | 12 | 112059557 | T | C | 0.37 | 0.068 | 0.010 | 1.93E-10 | 0.0003 | 46 |  |  |  |
| CAD | rs2681472 | 12 | 90008959 | G | A | 0.20 | 0.077 | 0.012 | 6.17E-11 | 0.0002 | 41 |  |  |  |
| CAD | rs11617955 | 13 | 110818102 | T | A | 0.89 | 0.086 | 0.016 | 3.55E-08 | 0.0002 | 29 |  |  |  |
| CAD | rs11838776 | 13 | 111040681 | A | G | 0.26 | 0.068 | 0.010 | 1.83E-10 | 0.0003 | 46 |  |  |  |
| CAD | rs10139550 | 14 | 100145710 | G | C | 0.42 | 0.058 | 0.010 | 1.38E-08 | 0.0002 | 34 |  |  |  |
| CAD | rs56062135 | 15 | 67455630 | C | T | 0.79 | 0.068 | 0.012 | 4.52E-09 | 0.0002 | 32 |  |  |  |
| CAD | rs8042271 | 15 | 89574218 | G | A | 0.90 | 0.095 | 0.019 | 3.68E-08 | 0.0001 | 25 |  |  |  |
| CAD | rs4468572 | 15 | 79124475 | C | T | 0.59 | 0.077 | 0.009 | 4.44E-16 | 0.0004 | 73 |  |  |  |
| CAD | rs7212798 | 17 | 59013488 | C | T | 0.15 | 0.077 | 0.014 | 1.88E-08 | 0.0002 | 30 |  |  |  |
| CAD | rs663129 | 18 | 57838401 | A | G | 0.26 | 0.058 | 0.010 | 3.20E-08 | 0.0002 | 34 |  |  | Body mass index |
| CAD | rs56289821 | 19 | 11188247 | G | A | 0.90 | 0.131 | 0.016 | 4.44E-15 | 0.0004 | 67 |  |  |  |
| CAD | rs4420638 | 19 | 45422946 | G | A | 0.17 | 0.095 | 0.014 | 7.07E-11 | 0.0002 | 46 |  |  | Body mass index |
| CAD | rs17678683 | 2 | 145286559 | G | T | 0.09 | 0.095 | 0.016 | 3.00E-09 | 0.0002 | 35 |  |  |  |
| CAD | rs16986953 | 2 | 19942473 | A | G | 0.10 | 0.086 | 0.014 | 1.45E-08 | 0.0002 | 38 |  |  |  |
| CAD | rs7568458 | 2 | 85788175 | A | T | 0.45 | 0.058 | 0.010 | 3.62E-10 | 0.0002 | 34 |  |  |  |
| CAD | rs192011340 | 2 | 21378433 | D | I | 0.75 | 0.068 | 0.014 | 2.89E-08 | 0.0001 | 24 | Not available |  |  |
| CAD | chr2:44074126 | 2 | 44074126 | I | D | 0.74 | 0.058 | 0.012 | 2.60E-08 | 0.0001 | 23 | Not available |  |  |
| CAD | rs201810558 | 2 | 203828796 | I | D | 0.11 | 0.140 | 0.016 | 2.15E-18 | 0.0004 | 77 | Not available |  |  |
| CAD | rs28451064 | 21 | 35593827 | A | G | 0.12 | 0.131 | 0.016 | 1.33E-15 | 0.0004 | 67 |  |  |  |
| CAD | rs180803 | 22 | 24658858 | G | T | 0.97 | 0.182 | 0.030 | 1.64E-10 | 0.0002 | 37 |  |  |  |
| CAD | rs377727514 | 3 | 138099161 | I | D | 0.16 | 0.077 | 0.012 | 2.89E-09 | 0.0002 | 41 |  | r^2^ > 0.01 |  |
| CAD | rs4593108 | 4 | 148281001 | C | G | 0.80 | 0.068 | 0.012 | 8.82E-10 | 0.0002 | 32 |  |  |  |
| CAD | rs72689147 | 4 | 156639888 | G | T | 0.82 | 0.068 | 0.012 | 6.07E-09 | 0.0002 | 32 |  | r^2^ > 0.01 |  |
| CAD | rs17087335 | 4 | 57838583 | T | G | 0.21 | 0.058 | 0.012 | 4.59E-08 | 0.0001 | 23 |  |  |  |
| CAD | rs9349379 | 6 | 12903957 | G | A | 0.43 | 0.131 | 0.009 | 1.81E-42 | 0.0011 | 212 |  |  |  |
| CAD | rs55730499 | 6 | 161005610 | T | C | 0.06 | 0.315 | 0.024 | 5.39E-39 | 0.0009 | 172 |  |  |  |
| CAD | rs2315065 | 6 | 161108144 | A | C | 0.06 | 0.285 | 0.025 | 2.88E-34 | 0.0007 | 130 |  | r^2^ > 0.01 |  |
| CAD | rs56336142 | 6 | 39134099 | T | C | 0.81 | 0.068 | 0.012 | 1.85E-08 | 0.0002 | 32 |  |  |  |
| CAD | rs11556924 | 7 | 129663496 | C | T | 0.69 | 0.077 | 0.012 | 5.34E-11 | 0.0002 | 41 |  |  |  |
| CAD | rs3918226 | 7 | 150690176 | T | C | 0.06 | 0.131 | 0.022 | 1.69E-09 | 0.0002 | 35 |  |  |  |
| CAD | rs2107595 | 7 | 19049388 | A | G | 0.20 | 0.077 | 0.012 | 8.05E-11 | 0.0002 | 41 |  |  |  |
| CAD | rs2519093 | 9 | 136141870 | T | C | 0.19 | 0.077 | 0.012 | 1.19E-11 | 0.0002 | 41 |  |  |  |
| CAD | rs2891168 | 9 | 22098619 | G | A | 0.49 | 0.191 | 0.010 | 2.29E-98 | 0.0020 | 365 |  |  |  |
| MI | rs35700460 | 1 | 222811407 | G | A | 0.65 | 0.086 | 0.012 | 1.88E-11 | 0.0003 | 51 |  |  |  |
| MI | rs7528419 | 1 | 109817192 | A | G | 0.80 | 0.104 | 0.012 | 9.71E-16 | 0.0005 | 75 |  |  |  |
| MI | rs9970807 | 1 | 56965664 | C | T | 0.92 | 0.113 | 0.018 | 1.79E-09 | 0.0002 | 39 |  |  |  |
| MI | rs10176176 | 2 | 85762048 | T | A | 0.47 | 0.068 | 0.010 | 2.88E-10 | 0.0003 | 46 |  |  |  |
| MI | rs201810558 | 2 | 203828796 | I | D | 0.11 | 0.131 | 0.016 | 5.47E-14 | 0.0004 | 67 | Not available |  |  |
| MI | rs72689147 | 4 | 156639888 | G | T | 0.82 | 0.077 | 0.012 | 1.63E-08 | 0.0002 | 41 |  |  |  |
| MI | rs10455872 | 6 | 161010118 | G | A | 0.05 | 0.285 | 0.025 | 8.88E-27 | 0.0008 | 130 |  |  |  |
| MI | rs1544935 | 6 | 39124448 | T | G | 0.81 | 0.077 | 0.012 | 2.89E-08 | 0.0002 | 41 |  |  |  |
| MI | rs2315065 | 6 | 161108144 | A | C | 0.06 | 0.262 | 0.024 | 2.34E-24 | 0.0007 | 119 |  | r^2^ > 0.01 |  |
| MI | rs9349379 | 6 | 12903957 | G | A | 0.41 | 0.131 | 0.009 | 8.90E-35 | 0.0013 | 212 |  |  |  |
| MI | rs11556924 | 7 | 129663496 | C | T | 0.70 | 0.068 | 0.012 | 3.96E-08 | 0.0002 | 32 |  |  |  |
| MI | rs2891168 | 9 | 22098619 | G | A | 0.48 | 0.191 | 0.008 | 5.03E-75 | 0.0034 | 570 |  |  |  |
| MI | rs532436 | 9 | 136149830 | A | G | 0.19 | 0.113 | 0.011 | 2.31E-17 | 0.0006 | 106 |  |  |  |
| MI | rs1332329 | 10 | 91003419 | C | A | 0.36 | 0.077 | 0.009 | 2.64E-13 | 0.0004 | 73 |  |  |  |
| MI | rs1870634 | 10 | 44480811 | G | T | 0.62 | 0.068 | 0.010 | 8.23E-11 | 0.0003 | 46 |  |  |  |
| MI | rs2505083 | 10 | 30335122 | C | T | 0.39 | 0.058 | 0.010 | 6.85E-09 | 0.0002 | 34 |  |  |  |
| MI | rs2019090 | 11 | 103668962 | A | T | 0.36 | 0.068 | 0.010 | 3.60E-09 | 0.0003 | 46 |  |  |  |
| MI | rs2681472 | 12 | 90008959 | G | A | 0.19 | 0.077 | 0.012 | 6.03E-09 | 0.0002 | 41 |  |  |  |
| MI | rs653178 | 12 | 112007756 | C | T | 0.44 | 0.077 | 0.009 | 2.80E-11 | 0.0004 | 73 |  |  | Body mass index |
| MI | rs7165042 | 15 | 79123338 | C | T | 0.56 | 0.058 | 0.012 | 3.02E-09 | 0.0001 | 23 |  |  |  |
| MI | rs55791371 | 19 | 11188153 | A | C | 0.90 | 0.104 | 0.018 | 2.95E-08 | 0.0002 | 33 |  |  |  |
| MI | rs56131196 | 19 | 45422846 | A | G | 0.16 | 0.086 | 0.014 | 3.87E-08 | 0.0002 | 38 |  |  | Body mass index |
| MI | rs28451064 | 21 | 35593827 | A | G | 0.12 | 0.122 | 0.018 | 6.00E-12 | 0.0003 | 46 |  |  |  |
| MI | rs180803 | 22 | 24658858 | G | T | 0.97 | 0.191 | 0.030 | 3.62E-09 | 0.0002 | 41 |  |  |  |
| Abbreviation: SNP, single nucleotide polymorphism; CAD, coronary artery disease; MI, myocardial infarction; MR, Mendelian randomization; Chr, chromosome; EA, effect allele; NEA, non-effect allele; EAF, effect allele frequency; SE, standard error.  ^a^ R^2^ were calculated using the following formula: (2×EAF×(1-EAF)×beta^2^)/[(2×EAF×(1-EAF)×beta^2^) + (2×EAF×(1-EAF)×N×SE^2^)], where EAF is the effect allele frequency, beta is the estimated effect on exposure, Ν is the sample size of the GWAS for the SNP-exposure association and SE is the standard error of the estimated effect.  ^b^ F statistic were calculated using the following formula: R^2^(N-2)/(1-R^2^), where R^2^ is the proportion of variance in exposure explained by each instrument and N is the sample size of the GWAS for the SNP-exposure association.  ^c^ SNPs which were not available in the outcome GWAS were replaced by their proxy (r^2^ >0.8) or deleted if the proxy was also not available.  ^d^ SNPs with r^2^ > 0.01 were removed.  ^e^ SNPs associated with confounding factors were removed after searching Phenoscanner database. | | | | | | | | | | | | | | |

| Supplementary Table S4 Genetic association of genetic variants with OA and its other phenotypes | | | | | | | | | | | | | | | | | | | | | |  |
| --- | --- | --- | --- | --- | --- | --- | --- | --- | --- | --- | --- | --- | --- | --- | --- | --- | --- | --- | --- | --- | --- | --- |
| Exposure | SNP | EA | NEA | Total OA | | | Knee OA | | | Hip OA | | | Spine OA | | | Hand OA | | | Thumb OA | | |  |
|  |  |  |  | Beta | SE | *P* value | Beta | SE | *P* value | Beta | SE | *P* value | Beta | SE | *P* value | Beta | SE | *P* value | Beta | SE | *P* value |  |
| CAD | rs7528419 | A | G | -0.014 | 0.005 | 0.01 | -0.006 | 0.008 | 0.50 | -0.039 | 0.011 | 0.00 | -0.012 | 0.012 | 0.33 | 0.024 | 0.015 | 0.10 | 0.007 | 0.020 | 0.73 |  |
| CAD | rs6689306 | A | G | -0.001 | 0.005 | 0.83 | -0.007 | 0.007 | 0.37 | -0.016 | 0.009 | 0.08 | -0.011 | 0.010 | 0.27 | 0.026 | 0.012 | 0.04 | 0.036 | 0.017 | 0.03 |  |
| CAD | rs67180937 | G | T | -0.008 | 0.006 | 0.17 | -0.012 | 0.010 | 0.23 | -0.003 | 0.012 | 0.79 | -0.023 | 0.014 | 0.11 | -0.012 | 0.017 | 0.50 | -0.019 | 0.019 | 0.33 |  |
| CAD | rs11206510 | T | C | 0.003 | 0.006 | 0.64 | -0.002 | 0.009 | 0.86 | 0.002 | 0.012 | 0.85 | -0.010 | 0.013 | 0.47 | -0.002 | 0.016 | 0.88 | -0.017 | 0.022 | 0.44 |  |
| CAD | rs9970807 | C | T | -0.005 | 0.008 | 0.52 | -0.002 | 0.012 | 0.89 | -0.012 | 0.016 | 0.46 | -0.018 | 0.018 | 0.32 | -0.016 | 0.022 | 0.46 | -0.026 | 0.030 | 0.40 |  |
| CAD | rs2487928 | A | G | -0.001 | 0.005 | 0.77 | 0.008 | 0.007 | 0.26 | 0.008 | 0.009 | 0.35 | -0.002 | 0.010 | 0.86 | -0.006 | 0.012 | 0.64 | -0.011 | 0.017 | 0.52 |  |
| CAD | rs1870634 | G | T | -0.002 | 0.005 | 0.63 | 0.001 | 0.008 | 0.89 | -0.006 | 0.010 | 0.55 | 0.002 | 0.010 | 0.85 | -0.007 | 0.013 | 0.57 | 0.038 | 0.018 | 0.03 |  |
| CAD | rs1412444 | T | C | -0.006 | 0.005 | 0.24 | -0.003 | 0.007 | 0.67 | -0.004 | 0.009 | 0.66 | -0.007 | 0.011 | 0.54 | -0.019 | 0.013 | 0.13 | -0.027 | 0.017 | 0.12 |  |
| CAD | rs2128739 | A | C | -0.002 | 0.005 | 0.63 | -0.004 | 0.008 | 0.63 | 0.005 | 0.010 | 0.61 | -0.016 | 0.011 | 0.15 | 0.005 | 0.013 | 0.72 | 0.011 | 0.019 | 0.56 |  |
| CAD | rs10840293 | A | G | -0.012 | 0.005 | 0.01 | -0.022 | 0.007 | 0.00 | -0.001 | 0.010 | 0.93 | -0.001 | 0.010 | 0.95 | -0.013 | 0.013 | 0.31 | -0.013 | 0.017 | 0.43 |  |
| CAD | rs11065979 | T | C | -0.004 | 0.005 | 0.45 | -0.021 | 0.007 | 0.00 | 0.000 | 0.018 | 1.00 | 0.001 | 0.010 | 0.93 | -0.018 | 0.012 | 0.15 | -0.014 | 0.017 | 0.43 |  |
| CAD | rs2681472 | G | A | 0.012 | 0.006 | 0.05 | 0.001 | 0.009 | 0.88 | 0.035 | 0.012 | 0.00 | -0.006 | 0.013 | 0.66 | 0.016 | 0.016 | 0.31 | 0.022 | 0.022 | 0.31 |  |
| CAD | rs11617955 | T | A | -0.005 | 0.007 | 0.49 | 0.001 | 0.012 | 0.94 | 0.001 | 0.015 | 0.95 | -0.001 | 0.016 | 0.95 | 0.012 | 0.019 | 0.52 | 0.004 | 0.025 | 0.89 |  |
| CAD | rs11838776 | A | G | -0.005 | 0.005 | 0.37 | -0.003 | 0.008 | 0.75 | -0.003 | 0.010 | 0.80 | -0.004 | 0.011 | 0.71 | 0.007 | 0.013 | 0.63 | 0.032 | 0.018 | 0.08 |  |
| CAD | rs10139550 | G | C | -0.004 | 0.005 | 0.40 | -0.012 | 0.007 | 0.10 | -0.002 | 0.009 | 0.86 | 0.014 | 0.010 | 0.17 | -0.010 | 0.012 | 0.43 | -0.008 | 0.017 | 0.64 |  |
| CAD | rs56062135 | C | T | -0.011 | 0.005 | 0.05 | -0.010 | 0.008 | 0.21 | -0.010 | 0.011 | 0.34 | -0.016 | 0.012 | 0.19 | -0.014 | 0.014 | 0.34 | 0.005 | 0.020 | 0.80 |  |
| CAD | rs8042271 | G | A | 0.003 | 0.011 | 0.81 | 0.007 | 0.017 | 0.69 | 0.012 | 0.023 | 0.59 | 0.025 | 0.023 | 0.28 | 0.000 | 0.031 | 0.99 | -0.032 | 0.042 | 0.45 |  |
| CAD | rs4468572 | C | T | 0.012 | 0.005 | 0.01 | 0.015 | 0.007 | 0.04 | 0.016 | 0.009 | 0.08 | 0.012 | 0.010 | 0.23 | 0.026 | 0.012 | 0.04 | 0.013 | 0.017 | 0.46 |  |
| CAD | rs7212798 | C | T | 0.016 | 0.008 | 0.04 | 0.029 | 0.015 | 0.04 | 0.007 | 0.018 | 0.68 | -0.005 | 0.019 | 0.79 | 0.001 | 0.023 | 0.98 | 0.005 | 0.028 | 0.85 |  |
| CAD | rs56289821 | G | A | -0.012 | 0.007 | 0.11 | -0.010 | 0.011 | 0.37 | -0.022 | 0.014 | 0.13 | -0.010 | 0.016 | 0.53 | -0.026 | 0.020 | 0.19 | -0.013 | 0.028 | 0.63 |  |
| CAD | rs17678683 | G | T | 0.015 | 0.008 | 0.06 | 0.012 | 0.012 | 0.32 | -0.012 | 0.016 | 0.46 | 0.049 | 0.017 | 0.00 | 0.031 | 0.020 | 0.12 | 0.022 | 0.026 | 0.40 |  |
| CAD | rs16986953 | A | G | -0.001 | 0.009 | 0.89 | -0.001 | 0.014 | 0.95 | -0.021 | 0.018 | 0.24 | 0.021 | 0.019 | 0.26 | 0.023 | 0.024 | 0.35 | 0.019 | 0.034 | 0.57 |  |
| CAD | rs7568458 | A | T | 0.005 | 0.005 | 0.29 | 0.004 | 0.007 | 0.57 | 0.020 | 0.009 | 0.03 | 0.002 | 0.010 | 0.88 | 0.014 | 0.012 | 0.25 | 0.006 | 0.017 | 0.73 |  |
| CAD | rs28451064 | A | G | -0.008 | 0.007 | 0.26 | -0.016 | 0.011 | 0.14 | 0.014 | 0.013 | 0.29 | -0.025 | 0.015 | 0.10 | -0.015 | 0.018 | 0.42 | 0.019 | 0.025 | 0.45 |  |
| CAD | rs180803 | G | T | -0.007 | 0.025 | 0.78 | 0.019 | 0.043 | 0.66 | -0.089 | 0.057 | 0.12 | -0.042 | 0.057 | 0.46 | -0.014 | 0.074 | 0.85 | 0.024 | 0.085 | 0.77 |  |
| CAD | rs4593108 | C | G | 0.008 | 0.006 | 0.16 | 0.010 | 0.009 | 0.29 | -0.005 | 0.012 | 0.65 | 0.018 | 0.013 | 0.17 | 0.016 | 0.016 | 0.32 | -0.006 | 0.022 | 0.78 |  |
| CAD | rs17087335 | T | G | -0.005 | 0.006 | 0.38 | 0.026 | 0.009 | 0.00 | -0.032 | 0.011 | 0.00 | -0.002 | 0.012 | 0.85 | 0.018 | 0.015 | 0.23 | -0.008 | 0.021 | 0.69 |  |
| CAD | rs9349379 | G | A | -0.013 | 0.005 | 0.01 | -0.011 | 0.007 | 0.12 | -0.017 | 0.009 | 0.07 | -0.017 | 0.010 | 0.09 | 0.016 | 0.012 | 0.18 | 0.015 | 0.017 | 0.36 |  |
| CAD | rs55730499 | T | C | -0.014 | 0.009 | 0.13 | -0.024 | 0.014 | 0.09 | -0.027 | 0.018 | 0.13 | -0.026 | 0.020 | 0.20 | -0.042 | 0.025 | 0.10 | -0.039 | 0.034 | 0.25 |  |
| CAD | rs56336142 | T | C | -0.002 | 0.006 | 0.74 | 0.007 | 0.009 | 0.41 | 0.007 | 0.011 | 0.53 | 0.007 | 0.012 | 0.55 | -0.017 | 0.015 | 0.24 | -0.022 | 0.020 | 0.29 |  |
| CAD | rs11556924 | C | T | -0.007 | 0.005 | 0.14 | -0.008 | 0.007 | 0.25 | 0.008 | 0.009 | 0.40 | -0.019 | 0.010 | 0.07 | 0.011 | 0.013 | 0.37 | 0.003 | 0.017 | 0.88 |  |
| CAD | rs3918226 | T | C | -0.023 | 0.008 | 0.01 | -0.037 | 0.013 | 0.00 | -0.010 | 0.016 | 0.54 | -0.026 | 0.019 | 0.17 | -0.011 | 0.022 | 0.61 | -0.047 | 0.030 | 0.12 |  |
| CAD | rs2107595 | A | G | 0.017 | 0.006 | 0.01 | 0.028 | 0.010 | 0.00 | 0.009 | 0.012 | 0.46 | 0.018 | 0.013 | 0.18 | 0.028 | 0.016 | 0.09 | -0.001 | 0.024 | 0.98 |  |
| CAD | rs2519093 | T | C | 0.002 | 0.006 | 0.68 | 0.019 | 0.009 | 0.03 | -0.021 | 0.012 | 0.06 | 0.002 | 0.012 | 0.88 | 0.023 | 0.016 | 0.14 | 0.018 | 0.023 | 0.42 |  |
| CAD | rs2891168 | G | A | -0.008 | 0.005 | 0.06 | -0.005 | 0.007 | 0.47 | -0.004 | 0.009 | 0.68 | -0.001 | 0.010 | 0.89 | -0.009 | 0.012 | 0.48 | -0.009 | 0.017 | 0.59 |  |
| MI | rs35700460 | G | A | -0.008 | 0.005 | 0.12 | -0.017 | 0.008 | 0.04 | 0.017 | 0.009 | 0.06 | -0.017 | 0.011 | 0.13 | -0.016 | 0.014 | 0.25 | -0.017 | 0.019 | 0.37 |  |
| MI | rs7528419 | A | G | -0.014 | 0.005 | 0.01 | -0.006 | 0.008 | 0.50 | -0.025 | 0.018 | 0.16 | -0.012 | 0.012 | 0.33 | 0.024 | 0.015 | 0.10 | 0.007 | 0.020 | 0.73 |  |
| MI | rs9970807 | C | T | -0.005 | 0.008 | 0.52 | -0.002 | 0.012 | 0.89 | 0.008 | 0.009 | 0.40 | -0.018 | 0.018 | 0.32 | -0.016 | 0.022 | 0.46 | -0.026 | 0.030 | 0.40 |  |
| MI | rs10176176 | T | A | 0.006 | 0.005 | 0.23 | 0.001 | 0.007 | 0.88 | -0.005 | 0.009 | 0.60 | 0.003 | 0.010 | 0.75 | 0.018 | 0.012 | 0.15 | 0.012 | 0.017 | 0.46 |  |
| MI | rs72689147 | G | T | 0.005 | 0.006 | 0.39 | 0.002 | 0.009 | 0.79 | 0.005 | 0.011 | 0.65 | -0.007 | 0.013 | 0.58 | 0.015 | 0.016 | 0.34 | 0.027 | 0.022 | 0.21 |  |
| MI | rs10455872 | G | A | -0.013 | 0.009 | 0.14 | -0.023 | 0.014 | 0.11 | -0.089 | 0.057 | 0.12 | -0.026 | 0.020 | 0.20 | -0.039 | 0.025 | 0.12 | -0.037 | 0.034 | 0.28 |  |
| MI | rs1544935 | T | G | -0.002 | 0.006 | 0.74 | 0.008 | 0.009 | 0.37 | -0.006 | 0.010 | 0.55 | 0.004 | 0.012 | 0.75 | -0.020 | 0.015 | 0.19 | -0.024 | 0.020 | 0.23 |  |
| MI | rs9349379 | G | A | -0.013 | 0.005 | 0.01 | -0.011 | 0.007 | 0.12 | 0.005 | 0.010 | 0.60 | -0.017 | 0.010 | 0.09 | 0.016 | 0.012 | 0.18 | 0.015 | 0.017 | 0.36 |  |
| MI | rs11556924 | C | T | -0.007 | 0.005 | 0.14 | -0.008 | 0.007 | 0.25 | 0.008 | 0.009 | 0.39 | -0.019 | 0.010 | 0.07 | 0.011 | 0.013 | 0.37 | 0.003 | 0.017 | 0.88 |  |
| MI | rs2891168 | G | A | -0.008 | 0.005 | 0.06 | -0.005 | 0.007 | 0.47 | 0.035 | 0.012 | 0.00 | -0.001 | 0.010 | 0.89 | -0.009 | 0.012 | 0.48 | -0.009 | 0.017 | 0.59 |  |
| MI | rs532436 | A | G | 0.003 | 0.006 | 0.62 | 0.020 | 0.009 | 0.03 | 0.014 | 0.013 | 0.29 | 0.002 | 0.012 | 0.87 | 0.024 | 0.016 | 0.13 | 0.020 | 0.023 | 0.38 |  |
| MI | rs1332329 | C | A | -0.006 | 0.005 | 0.20 | -0.002 | 0.007 | 0.84 | -0.004 | 0.009 | 0.68 | -0.009 | 0.011 | 0.39 | -0.017 | 0.013 | 0.19 | -0.022 | 0.017 | 0.20 |  |
| MI | rs1870634 | G | T | -0.002 | 0.005 | 0.63 | 0.001 | 0.008 | 0.89 | -0.009 | 0.010 | 0.37 | 0.002 | 0.010 | 0.85 | -0.007 | 0.013 | 0.57 | 0.038 | 0.018 | 0.03 |  |
| MI | rs2505083 | C | T | 0.001 | 0.005 | 0.76 | 0.007 | 0.007 | 0.36 | -0.021 | 0.012 | 0.08 | -0.003 | 0.010 | 0.78 | -0.014 | 0.012 | 0.26 | -0.022 | 0.017 | 0.20 |  |
| MI | rs2019090 | A | T | -0.003 | 0.005 | 0.61 | -0.001 | 0.008 | 0.86 | -0.022 | 0.014 | 0.13 | -0.016 | 0.011 | 0.15 | 0.006 | 0.013 | 0.64 | 0.013 | 0.019 | 0.49 |  |
| MI | rs2681472 | G | A | 0.012 | 0.006 | 0.05 | 0.001 | 0.009 | 0.88 | -0.016 | 0.009 | 0.09 | -0.006 | 0.013 | 0.66 | 0.016 | 0.016 | 0.31 | 0.022 | 0.022 | 0.31 |  |
| MI | rs7165042 | C | T | -0.012 | 0.005 | 0.01 | -0.015 | 0.007 | 0.04 | 0.007 | 0.012 | 0.52 | -0.012 | 0.010 | 0.23 | -0.025 | 0.012 | 0.04 | -0.013 | 0.017 | 0.46 |  |
| MI | rs55791371 | A | C | -0.012 | 0.007 | 0.11 | -0.010 | 0.011 | 0.36 | -0.039 | 0.011 | 0.00 | -0.010 | 0.016 | 0.52 | -0.028 | 0.020 | 0.16 | -0.014 | 0.028 | 0.61 |  |
| MI | rs28451064 | A | G | -0.008 | 0.007 | 0.26 | -0.016 | 0.011 | 0.14 | -0.017 | 0.009 | 0.07 | -0.025 | 0.015 | 0.10 | -0.015 | 0.018 | 0.42 | 0.019 | 0.025 | 0.45 |  |
| MI | rs180803 | G | T | -0.007 | 0.025 | 0.78 | 0.019 | 0.043 | 0.66 | -0.012 | 0.016 | 0.46 | -0.042 | 0.057 | 0.46 | -0.014 | 0.074 | 0.85 | 0.024 | 0.085 | 0.77 |  |
| Abbreviation: OA, osteoarthritis; SNP, single nucleotide polymorphism; EA, effect allele; NEA, non-effect allele; SE, standard error; CAD, coronary artery disease; MI, myocardial infarction. | | | | | | | | | | | | | | | | | | | | | | |

Supplementary Figure S1 Scatter plots for myocardial infarction and total and spine OA

Plots show (A) the effect sizes of the SNP effects on total OA (y-axes) and the SNP effects on myocardial infarction (x-axes) with 95% confidence intervals, and (B) the effect sizes of the SNP effects on spine OA (y-axes) and the SNP effects on myocardial infarction (x-axes) with 95% confidence intervals. Each dot represents one of the 95 SNPs used in the myocardial infarction genetic instrument. The slopes indicate the estimate for each of the five different MR tests.


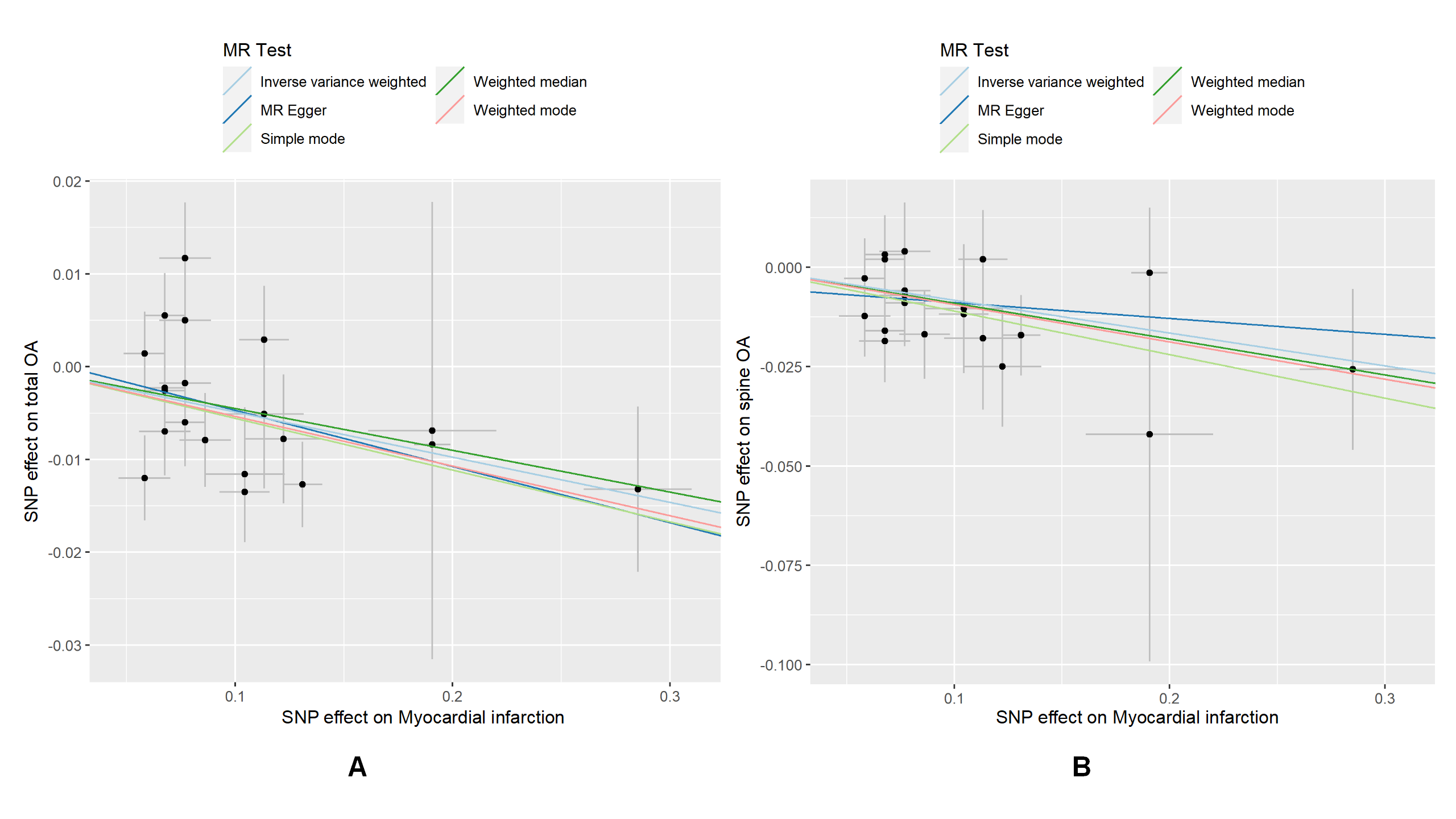


Supplementary Figure S2 Funnel plots for myocardial infarction and total and spine OA

(A) myocardial infarction and total OA. (B) myocardial infarction and spine OA. The plots show the effect estimate (b) of a particular SNP against the SNP expected precision (1/Standard Error (SE)). Asymmetry in the funnel plot is an indication of horizontal pleiotropy. The dark and light blue lines represent the MR-Egger and Inverse variance weighted slopes. respectively.
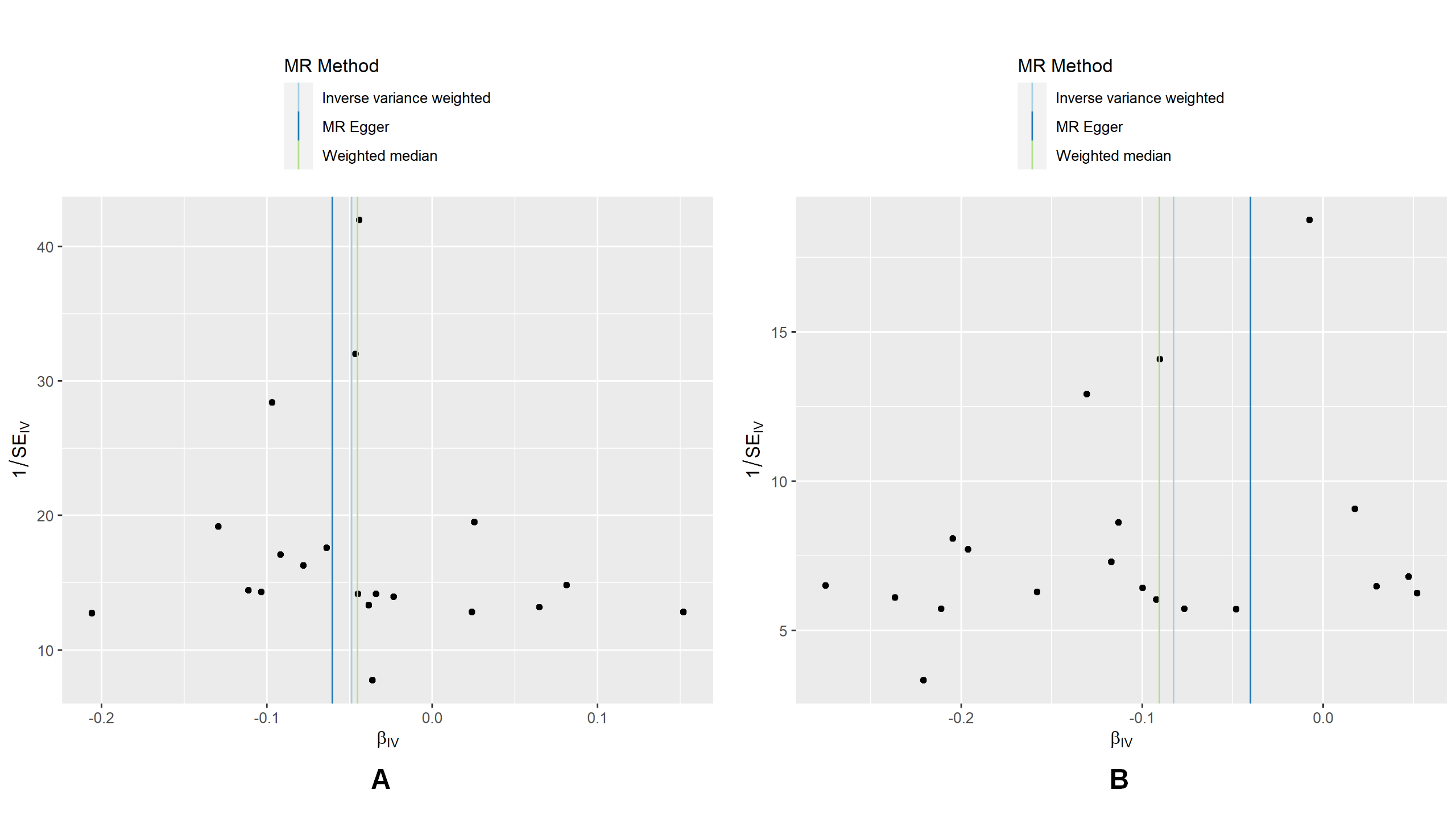


Supplementary Figure S3 Leave-one-out sensitivity analysis for myocardial infarction and total and spine OA

(A) myocardial infarction and total OA. (B) myocardial infarction and spine OA. The plots show the estimate effect by sequentially dropping one SNP at a time. Each black dot in the forest plot represents the MR results (IVW method) excluding that particular SNP. The result including all SNPs is shown in red at the bottom of the plot.


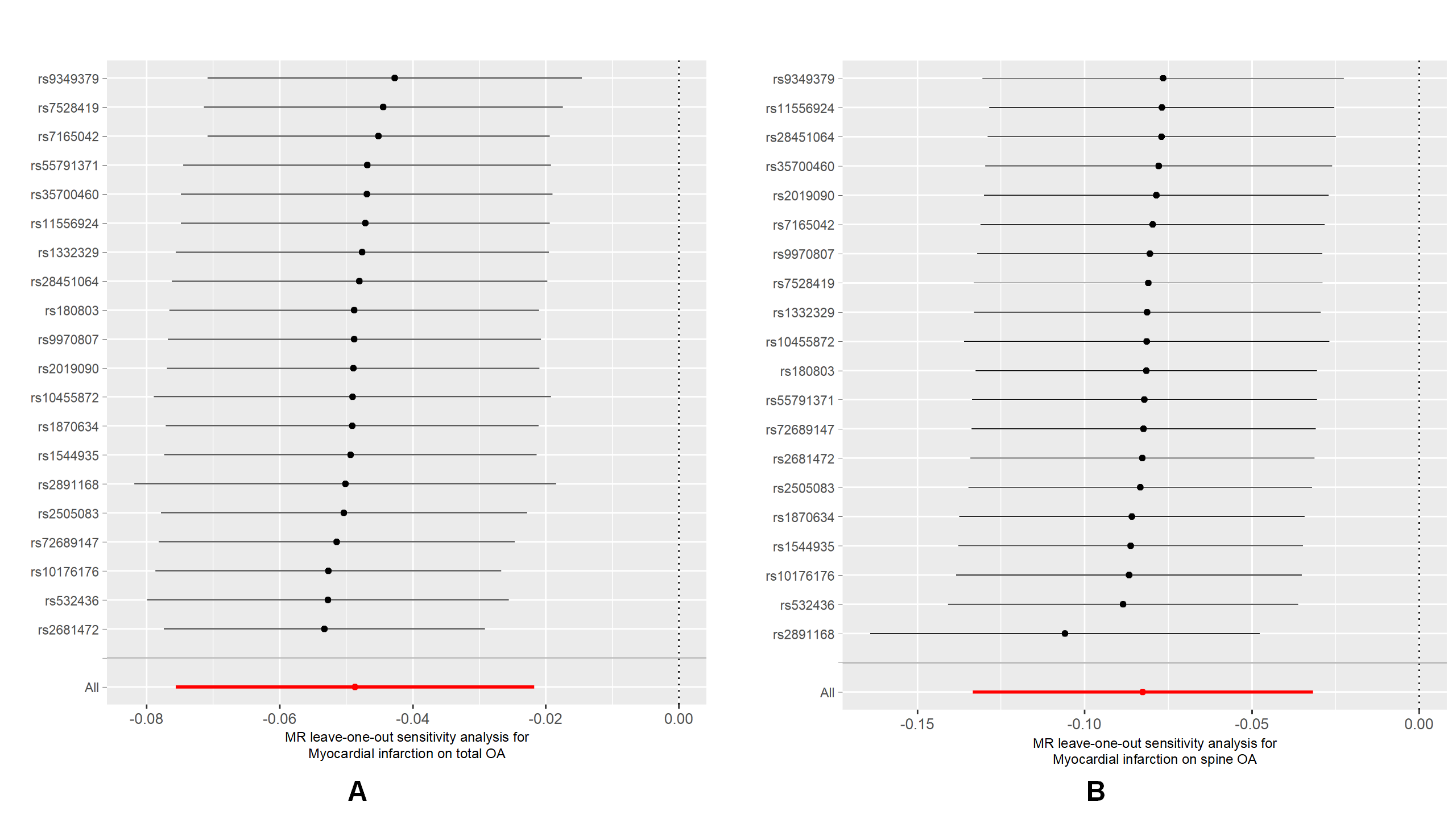

Supplement: Supplementary file 1 [file Data_Sheet_1.docx]
